# (Mg,Mn,Fe,Co,Ni)O: A rocksalt high-entropy oxide containing divalent Mn and Fe

**DOI:** 10.1126/sciadv.adi8809

**Published:** 2023-09-20

**Authors:** Yuguang Pu, Duncan Moseley, Zhen He, Krishna Chaitanya Pitike, Michael E. Manley, Jiaqiang Yan, Valentino R. Cooper, Valerie Mitchell, Vanessa K. Peterson, Bernt Johannessen, Raphael P. Hermann, Peng Cao

**Affiliations:** ^1^ Department of Chemical and Materials Engineering, University of Auckland, Private Bag 92019, Auckland 1142, New Zealand.; ^2^ Materials Science and Technology Division, Oak Ridge National Laboratory, Oak Ridge, TN 37831, USA.; ^3^ School of Materials Science and Engineering, Jiangsu University of Science and Technology, Zhenjiang 212003, China.; ^4^ Nuclear Sciences Division, Pacific Northwest National Laboratory, Richland, WA 99352, USA.; ^5^ Australian Synchrotron, Australian Nuclear Science and Technology Organisation, Clayton, VIC 3168, Australia.; ^6^ Australian Centre for Neutron Scattering, Australian Nuclear Science and Technology Organisation, Sydney, New South Wales 2232, Australia.; ^7^ MacDiarmid Institute for Advanced Materials and Nanotechnology, Victoria University Wellington, PO Box 600, Wellington, New Zealand.

## Abstract

High-entropy oxides (HEOs) have aroused growing interest due to fundamental questions relating to their structure formation, phase stability, and the interplay between configurational disorder and physical and chemical properties. Introducing Fe(II) and Mn(II) into a rocksalt HEO is considered challenging, as theoretical analysis suggests that they are unstable in this structure under ambient conditions. Here, we develop a bottom-up method for synthesizing Mn- and Fe-containing rocksalt HEO (FeO-HEO). We present a comprehensive investigation of its crystal structure and the random cation-site occupancy. We show the improved structural robustness of this FeO-HEO and verify the viability of an oxygen sublattice as a buffer layer. Compositional analysis reveals the valence and spin state of the iron species. We further report the antiferromagnetic order of this FeO-HEO below the transition temperature ~218 K and predict the conditions of phase stability of Mn- and Fe-containing HEOs. Our results provide fresh insights into the design and property tailoring of emerging classes of HEOs.

## Teaser

A generic method was developed to synthesize rocksalt high-entropy oxides containing divalent manganese and iron.

## INTRODUCTION

Since the successful fabrication of the first-ever entropy- stabilized oxides, or, more general, high-entropy oxides (HEOs), such solid solutions have drawn much attention due to their unique compositional and structural features, namely, the extreme chemical disorder incorporated into single-crystal structures (*1*–*3*). One representative example is that the metal ions in (Mg_0.2_Co_0.2_Ni_0.2_Cu_0.2_Zn_0.2_)O are unanimously divalent and randomly occupy the cationic sites in a rocksalt structure. These cations are coordinated by lattice oxygen in an octahedral configuration, even though Cu(II) and Zn(II) are usually four coordinated to oxygen atoms as, for example, in their binary oxides (*4*–*6*). This stabilization of Cu(II) and Zn(II) in an unfavorable configuration is ascribable to the increased configurational entropy and a high solid solution temperature (*1*, *7*, *8*).

Inspired by rocksalt HEOs, researchers have undertaken extensive work to develop diverse types of HEOs. HEOs in different crystal structures have been synthesized accordingly in the past decade, such as fluorite (*9*, *10*), perovskite (*11*–*13*), spinel (*14*–*16*), bixbyite (*17*), and pyrochlore (*18*, *19*). Further, a wide range of functional properties of the above HEOs, including electrochemical (*20*–*22*), catalytic (*23*–*25*), ionic conductivity (*26*, *27*), and magnetic properties (*13*, *28*, *29*), have been extensively explored. The chemical and physical properties stemming from the chemical complexity and unique crystal structures of HEOs have driven intense interest in this emerging class of ceramics (*30*–*33*).

Although the remarkable development of HEOs originates with the rocksalt type, studies on simple rocksalt HEOs are still based on the classic formulation (Mg,Co,Ni,Cu,Zn)O and its derivatives (*1*, *2*, *34*, *35*). Intriguingly, both Fe(II) and Mn(II) are octahedrally coordinated in their monoxides. Whether these ions can be stabilized in a rocksalt HEO becomes a rather interesting research question, in particular in light of theoretical predictions that suggest that Fe(II) and Mn(II) should not be stable in a rocksalt structure under ambient conditions (*36*). Previous studies also show that wüstite FeO is thermodynamically unstable at room temperature, as it spontaneously disproportionates into metallic Fe and Fe_3_O_4_ (*37*–*39*). All these facts render the extension of the classic formulation to other 3*d* transition metals a major challenge. Furthermore, while several studies have focused on the magnetic properties of Mn-/Fe-containing perovskite and spinel HEOs (*13*, *40*), there is a notable lack of relevant research into simple rocksalt HEOs containing Fe and Mn.

In this work, we present an original rocksalt HEO material, (Mg,Mn,Fe,Co,Ni)O. This HEO has a single-phase rocksalt structure, analogous to classic (Mg,Co,Ni,Cu,Zn)O. We further demonstrate that this material is a multicomponent solid solution as all constituent binary monoxides crystallize into a rocksalt-type structure, despite the nuances in lattice parameters. We also validate that in this structure, the cation site occupancy is highly random, and the metal ions incorporated in this compound are unanimously divalent. By using different in situ techniques, we demonstrate the improved structural robustness of this FeO-HEO compared to that of its constituent binary oxides. The short-range structure investigation reveals the functionality of an oxygen sublattice as a buffer layer to accommodate different metal ions. We subsequently evaluated the magnetic properties of this FeO-HEO, with magnetic susceptibility and heat capacity results revealing an antiferromagnetic (AFM) order with a magnetic transition temperature of ~218 K. Furthermore, the theoretical prediction suggests several different rocksalt HEOs can potentially be synthesized under low oxygen partial pressure. These fine FeO-HEO particles containing different 3*d* divalent ions are appealing as catalysts and energy storage materials (*30*, *41*). Its unique structure with extreme chemical disorder and a large magnetic moment is also interesting for research on structurally disordered magnetic materials.

## RESULTS

### Synthesis and characterizations of the FeO-HEO

**Fig. 1.**
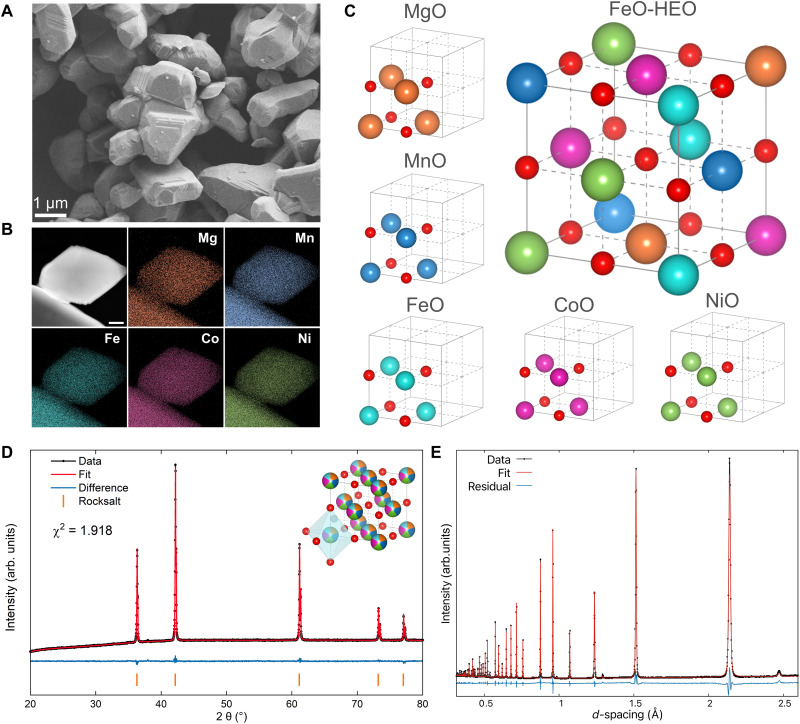
Characterizations and structural illustration of the FeO-HEO. (**A**) SEM image of the as-synthesized FeO-HEO. (**B**) DF-STEM image of the FeO-HEO and EDS maps of involved metal elements. Scale bar, 100 nm. (**C**) Schematic illustration of constituent binary oxides and the FeO-HEO product. The cation sites in the FeO-HEO are randomly occupied by different metal ions. Orange, blue, cyan, magenta, green, and red spheres represent Mg, Mn, Fe, Co, Ni, and O atoms, respectively. MgO [*F*m-3*m*, 4.214(1) Å, ICSD 52026], MnO [*F*m-3*m*, 4.446(1) Å, ICSD 9864], Fe_0.944_O [*F*m-3*m*, 4.263(1) Å, ICSD 27854], CoO (*F*m-3*m*, 4.263(1) Å, ICSD 9865), and NiO (*F*m-3*m*, 4.308(4) Å, ICSD 9866) are used as structural references. (**D**) XRD patterns with the refined crystal structure (inset). Orange, blue, cyan, magenta, green, and red spheres represent Mg, Mn, Fe, Co, Ni, and O atoms, respectively. (**E**) Rietveld refinement profiles for the rocksalt (Mg,Mn,Fe,Co,Ni)O using NPD data (POWGEN, SNS, at ORNL).

Our synthesis strategy of the precursors using oxalate anions as the “bridging” ligand is described in Materials and Methods, with additional details in the Supplementary Materials. Note that divalent cations like Fe(II) and Mn(II) are vulnerable to oxidation. Therefore, we conduct the synthesis of oxalate precursors in an inert atmosphere. The FeO-HEO can be obtained by annealing this precursor in an argon-filled tube furnace with the presence of a certain amount of MnO_2_ as the oxygen generator. The purpose of introducing MnO_2_ is to neutralize the reductive environment generated by the thermal decomposition of oxalate precursors (*42*), as MnO_2_ decomposes progressively at high temperatures and releases a small amount of O_2_ (*43*, *44*). As detailed in the Supplementary Materials, while an excess of generated oxygen leads to the formation of a spinel structure, insufficient oxygen introduction results in alloy formation. Hence, proper control of the oxygen generator quantity is key to obtaining single-phase FeO-HEO. The scanning electron microscopy (SEM) image (Fig. 1A) shows the good retention of the particle size of the precursor after annealing in argon. The observed particle sizes range from 500 nm to 3 μm. Figure 1B presents the dark-field scanning transmission electron microscopy (DF-STEM) analysis and energy-dispersive spectroscopy (EDS) elemental mapping of the FeO-HEO particles. Within the representative FeO-HEO particles, we observe a uniform distribution of different metal species.

Table 1 lists the structural information for the constituent binaries. The pristine crystals of these binary oxides have the same space group and unit-cell dimensions, despite some slight differences in lattice parameters (*45*, *46*). Thus, one can visualize this FeO-HEO as a solid solution in which cations of a parent oxide are proportionally substituted by similar cations without changing the nature of chemical bonding and crystal structure. Such a solid solution process coincides with the basic concept of isomorphous substitution, which is intuitively demonstrated in Fig. 1C (*47*).

**Table 1.** Structural information of related binary oxides.

Component	Space group	Lattice parameter (Å)	Source
MgO	*F*m-3*m*	4.217	(*45*)
MnO		4.446	(*45*)
FeO		4.309	(*46*)
CoO		4.263	(*45*)
NiO		4.178	(*45*)
FeO-HEO		4.2831(1) (POWGEN), 4.2818(1) (Echidna)	This work

The crystal structure of the FeO-HEO was studied using x-ray powder diffraction (XRD). The refinement results based on XRD shown in Fig. 1D indicate that this FeO-HEO has an ideal rocksalt structure. Even though the product was obtained by natural cooling to room temperature, we observed no phase segregation or peak broadening due to this slow cooling process from the XRD results (*48*). This phase compatibility of the FeO-HEO at low temperatures arises from the similar structures of constituent binary oxides. The inset in Fig. 1D displays that oxygen anions reside at 4*b* sites in the refined structure, while different metal ions occupy the octahedral 4*a* sites with a coordination number of 6.

As shown in table S2, these 3*d* transition metals have similar x-ray scattering cross sections, meaning that XRD is incapable of distinguishing adjacent elements, such as Mn, Fe, Co, and Ni (*49*). Hence, XRD results can only indicate a single-phase rocksalt structure but not ascertain the highly random site occupancy. We thus exploited high-resolution neutron powder diffraction (NPD) to verify the random cation site occupancy, since the involved metal atoms show notably different neutron coherent scattering cross sections (table S2). As shown in Fig. 1E, we refined a paramagnetic phase rocksalt cubic structure (*F*m*-*3*m*) model against the NPD data with mixed cation occupancy (that is, 20% for each), as in (*28*). The refined lattice parameter is *a* = 4.2831(1) Å at 300 K. The isotropic atomic displacement parameters *B*_iso_ are 0.475(15) and 0.612(15) Å^2^ for the metal (average) and oxygen atoms, respectively. These *B*_iso_ values are moderate compared with those of (Mg_0.2_Co_0.2_Ni_0.2_Cu_0.2_Zn_0.2_)O and some binaries (details in table S3). This fit produces a weighted profile residual *R*_wp_ of 10.4%. There is no observable extraneous or superstructure reflections or notable deviation in peak intensities. We, therefore, conclude that within our resolution (∆*d*/*d* ~ 0.0008), the sample is single phase, and there are no deviations from random occupancy of the cations.

### Phase stability

Because gradual oxidation of wüstite FeO nanoparticles into inverse-spinel Fe_3_O_4_ under ambient conditions has been widely observed (*39*, *50*, *51*), we evaluated the phase stability of this FeO-HEO in both oxidative and reductive atmospheres. We first analyzed its phase components by using NPD after exposing the FeO-HEO sample to air for more than 90 days. As shown in fig. S5, the results indicate no detectable phase segregation. In particular, no formation of a spinel phase can be observed in the diffraction pattern. The Rietveld refinement on these NPD results is well consistent with the refined structure of the fresh FeO-HEO, c.f. Fig. 1E.

**Fig. 2.**
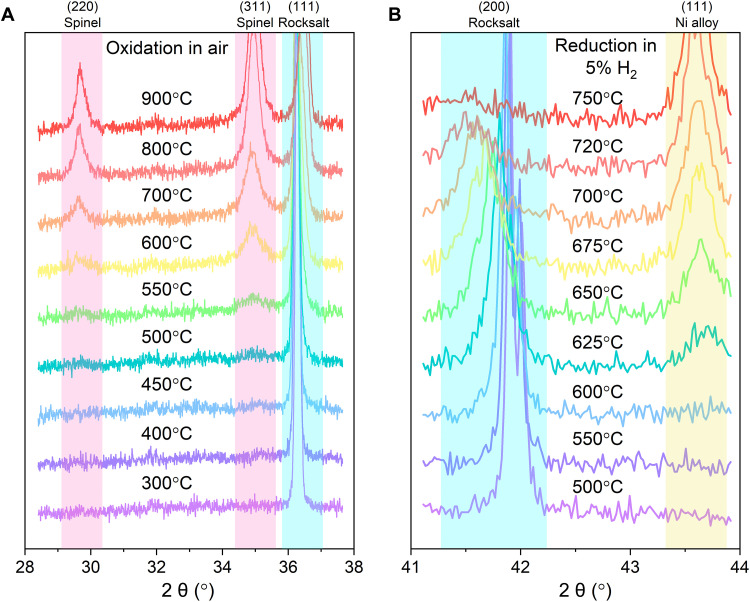
Evaluation of phase stability. (**A**) In situ XRD results of the FeO-HEO annealed in air. (**B**) In situ XRD results of the FeO-HEO reduced in 5% H_2_/Ar. Cyan regions identify diffraction peaks from rocksalt, while magenta and cream regions indicate peaks from spinel and metallic phases, respectively.

The phase transformation of the FeO-HEO from rocksalt to spinel was monitored by in situ XRD. The measurements were undertaken by annealing the sample in dry air at different temperatures for 10 min before collecting the XRD patterns. The XRD results acquired from 300° to 900°C are shown in Fig. 2A. Those peaks indexed to a spinel structure with a *Fd-*3*m* space group symmetry (magenta) become observable when the annealing temperature is above 450°C. The broadened (111) reflection of rocksalt (cyan), which shifts slightly to a higher diffraction angle, can be ascribed to the emergence of the (222) reflection of spinel. The reported conversion temperatures for both FeO and CoO nanoparticles are much lower than 450°C (*52*–*55*). This improved stability of divalent Fe and Co in FeO-HEO may be attributed to the larger particle size but also to the cocktail effect that is often observed for high-entropy alloys (*56*). Concretely, the properties of constituent binaries are greatly changed by the composition variation and solid-solution process.

We then used in situ XRD measurements to study the thermal reduction of FeO-HEO samples in 5% H_2_/Ar. As shown in Fig. 2B, the reduced metallic phase starts showing up when the reduction temperature exceeds 600°C. The (200) reflection of rocksalt (cyan) moves progressively to lower angles, which indicates larger lattice spacing, when the annealing temperature is elevated from 500°C to 750°C. One plausible explanation is the selective reduction of Ni and Co species from their oxide forms into metals. On the basis of the discussion about lattice parameters in Table 1, the removal of Ni and Co from the rocksalt structure of FeO-HEO will expand the lattice, given that the lattice parameters of some remaining oxides, such as MnO and FeO, are larger than that of FeO-HEO. Meanwhile, the selective reduction of homogeneously distributed Ni and Co ions from FeO-HEO breaks the long-range order of this crystal structure, thereby broadening the diffraction peaks indexed to the rocksalt structure. Note that the shoulder to the right side of the (200) diffraction peak is the Kα_2_ line.

The thermodynamic data in fig. S6 suggest that only NiO and CoO are reducible by hydrogen among these metal oxides within this temperature range, and NiO will be preferentially reduced. In accordance with previous studies, the reduction of pure NiO and CoO initiates at approximately 360°C and 400°C, respectively (*57*–*59*). However, Hu (*60*) reported that the reduction of Ni(II) and Co(II) in NiO-MgO and CoO-MgO solid solutions becomes more difficult because the formation of Ni-Ni (or Co-Co) bonds is conducive to the reduction of NiO (or CoO). The atomically dispersed Ni cations (or Co cations) are well isolated by nonreducible MgO in NiO-MgO (or CoO-MgO), which hampers the reduction of these metal ions (*60*). These findings are consistent with our experimental results. For example, the quasi in situ x-ray photoelectron spectroscopy (XPS) analysis in fig. S7 shows that the reduction of Ni occurs first at around 575°C, followed by the reduction of Co into a metallic state. We find no evidence of Fe and Mn metals forming on the FeO-HEO surface from the spectra. The gradual extension of Fe and Mn 2p spectra to lower binding energies implies that these two cations are gradually reduced to their subvalent states. Hence, the increased reduction temperatures for Ni and Co species in FeO-HEO, which is indicative of enhanced phase stability, could be due to the cocktail effect. In other words, Ni(II) and Co(II) become more difficult to be reduced by forming a homogeneous solid solution with less reducible cations, such as Fe(II), Mn(II), and Mg(II).

### Short-range structure and transition-metal environment

**Fig. 3.**
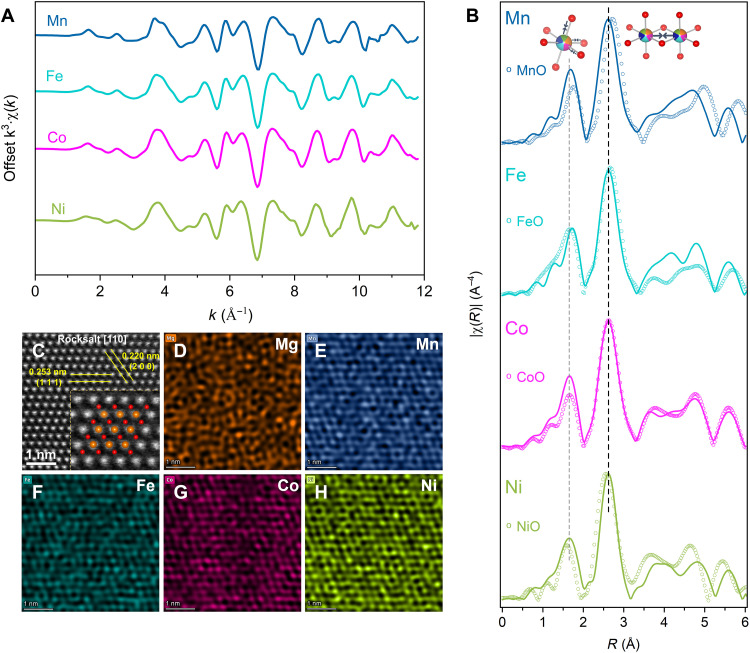
Short-range structure and chemical environment of the FeO-HEO. (**A**) EXAFS spectra in *k*-space for Mn, Fe, Co, and Ni absorbers in the FeO-HEO. Spectra are offset in *y* for clarity. (**B**) Fourier transforms of *k*-space oscillations for absorbers of interest in the FeO-HEO. Transforms are offset in *y* for clarity. Note that the intensities of the shells of FeO-HEO and binaries are normalized in y only for clarity and are not comparable. (**C**) HAADF image of the rocksalt structure of the FeO-HEO. The inset shows the atomic arrangement of the rocksalt structure along the [110] orientation. (**D** to **H**) EDS mappings of Mg, Mn, Fe, Co, and Ni.

To individually investigate the short-range structures surrounding Mn, Fe, Co, and Ni ions, we analyzed the extended x-ray absorption fine structure (EXAFS) of each absorber. The *K*-edge absorption energy of Mg is out of the energy range of the used beamline, making the technique not applicable. The raw spectra were calibrated using metal foils before converting them to the *k*-space spectra in Fig. 3A. Except for Ni, EXAFS data were truncated above 12 Å^−1^ to circumvent the interference from the following absorption edge. At a glance, the EXAFS data in *k*-space reveal that all these transition-metal absorbers (i.e., Mn, Fe, Co, and Ni) have similar short-range environments, indicating a homogeneous distribution of these cations in the lattice. In addition, the analogous amplitude and frequency of these oscillations imply a similar coordination geometry of these metal atoms (*4*). Unlike some local distortions observed in (Mg,Co,Ni,Cu,Zn)O (*1*, *4*), the similar scattering environments in FeO-HEO reflect an isostructural solid solution among MgO, MnO, FeO, CoO, and NiO.

Figure 3B shows the Fourier transforms of *k*^3^·χ(*k*) in Fig. 3A. Data in these transforms consistently have two prominent peaks in the radial distance range from 1 to 3 Å, which indicates the first two coordination shells in metal oxides. In detail, as demonstrated as the ball-stick models in Fig. 3B, the first intense peak between 1 and 2 Å and the second peak in the range of 2 to 3 Å correspond to the scattering between the center absorber and the first-near-neighbor oxygen atoms and the scattering between the center absorbers and the second-near-neighbor metal atoms, respectively. It is worth noting that there is a shift of about 0.5 Å in factual scattering distance as the phase shift in Fourier transform remains uncorrected. Intuitively, the distances from absorbers to their second-near-neighbor metal atoms are nearly identical, as evidenced by a similar peak position in all cases.

The Fourier transforms for all constituent binary oxides are superimposed on the corresponding transforms. Compared with the binary counterpart, both the first and second shells for Mn in the FeO-HEO shift to shorter scattering distances, while these shells for Ni in the FeO-HEO move far away from the absorbers. The nearest cation-to-anion and cation-to-cation distances remain unchanged for Co, as peaks in the transform for FeO-HEO show no shift toward the peaks for CoO. These changes in scattering distances are consistent with the difference in lattice parameters of constituent binary oxides in Table 1. More specifically, the Rietveld refinement based on NPD results suggests a unit cell parameter of 4.283 ± 0.002 Å, which is notably shorter than the lattice parameter of MnO but larger than that of NiO. Therefore, it can be understood that in this structure of FeO-HEO, lattices surrounding Mn and Fe are contracted compared with their primary lattices in MnO and FeO. In contrast, lattices surrounding Ni and Co are elongated compared to the lattices of their corresponding binary oxides. Because of the subtle differences among the lattice parameters of FeO, CoO, and the FeO-HEO, such shrinkage and expansion are negligible in the EXAFS results of Fe and Co absorbers. In practical terms, the Fourier transforms for Fe absorbers in FeO-HEO and FeO seem less reasonable, possibly due to the impure FeO reference material in which the Fe(II) is oxidized to a higher state.

These Fourier transforms of χ(*k*)⋅*k*^3^ for absorbers in FeO-HEO and binaries are overlapped and normalized in *y* for a clear demonstration of lattice shrinkage and expansion. A direct comparison between the intensities of those transforms of FeO-HEO and binaries in *y* axis is meaningless. However, the disagreement in the relative intensities of shells is informative. For FeO-HEO, the intensity ratio of the Co-O shell to the Co-Me shell (*I*_Co-O_/*I*_Co-Me_) is larger than the ratio for binary CoO. The increased relative intensity of the first coordination shells for some absorbers in FeO-HEO or the decrease in the relative intensity of the second shells (if we normalize the Me-O shells) is due to the formation of a solid solution between transition-metal oxides and MgO. According to the EXAFS equation, χ(*k*) depends on the atomic number *Z* of the scattering atom, meaning that heavy atoms show increased scattering amplitude (*61*). Thus, the dilution of transition-metal atoms in the second nearest coordination shells by Mg produces those slightly weak Me-Me shells. Similar phenomenon was observed for Co*_x_*Mg_1−*x*_O solid solutions by Kuzmin *et al. *(*62*).

Regardless of these Fourier transforms for binary oxides, the first coordination shells of all types of absorbers in the FeO-HEO are located at different radial distances, while the second nearest neighbor cation-to-cation distances are almost the same. These results agree well with the curve fitting for the first two coordination shells. As shown in fig. S8, the fits for absorbers were performed in the same window range, which is from 1 to 3 Å in Fourier transforms without phase correction. *R* factors for all curve fits are lower than 2%, suggesting that the fitting results are reliable. Fits for all absorbers used the crystal structure of CoO, because the structure parameters of the FeO-HEO are relatively close to those of CoO. Note that this structure model is an approximation without considering atomic numbers. Fitting results in table S4 show that the second nearest-neighbor cation-to-cation distances are nearly the same for all absorbers, while the first nearest-neighbor cation-to-anion bonds either shrink or expand depending on the types of center absorbers. The above results validate the function of the lattice oxygen as a buffer sublattice, enabling the accommodation of metal cations with different sizes and the retention of a scarcely distorted cation sublattice (*3*).

The FeO-HEO was also imaged using an aberration-corrected TEM to investigate the atomic arrangement and elemental distributions in an FeO-HEO particle. As shown in Fig. 3C, the STEM image reveals a highly ordered arrangement of different atoms along [110] orientation, matching well with the rocksalt model. The measured *d*-spacings for the (111) and (200) agree with the lattice distances obtained from XRD by considering the resolution of aberration-corrected TEM. The EDS mappings of Mg, Mn, Fe, Co, and Ni are exhibited in Fig. 3 (D to H) separately. It is explicit that different metal atoms are distributed randomly throughout the region of interest. No detectable cluster of any element is observed from the maps, suggesting a homogeneous dispersion of metal atoms in this solid solution.

### Compositional analysis

**Fig. 4.**
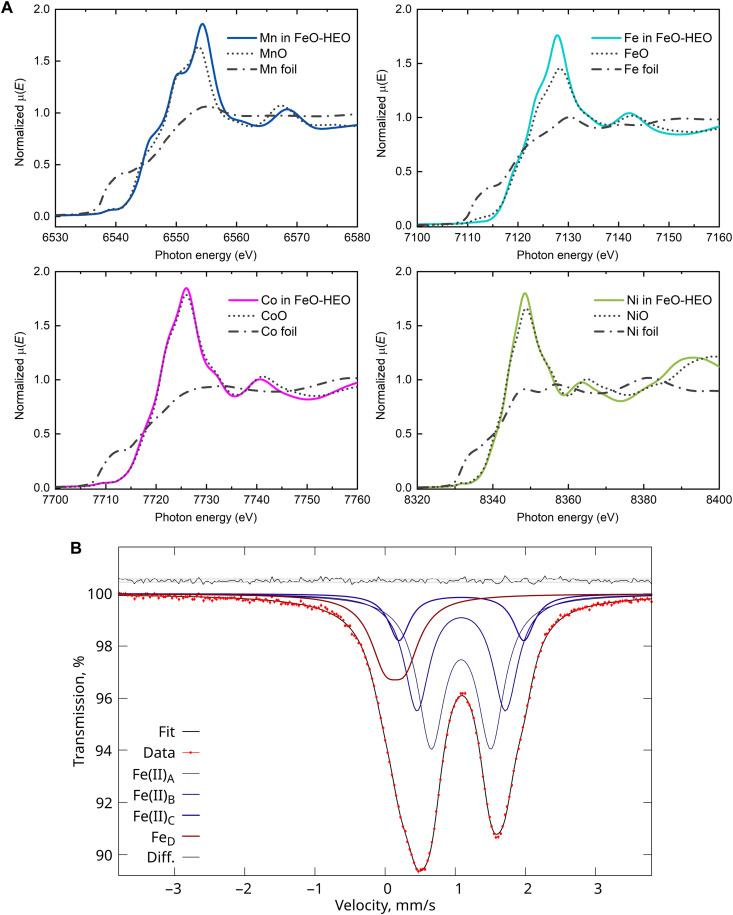
Compositional analysis of the FeO-HEO. (**A**) Mn-, Fe-, Co-, and Ni-*K* edges XANES spectra of corresponding absorbers in the FeO-HEO (solid), binary oxides (dotted), and metal foils (dot dash). (**B**) Fe-57 Mössbauer spectrum of the FeO-HEO recorded at room temperature. The four spectral components needed for a minimal fit that reproduces the data are indicated. Residuals and 1-σ interval (0.057%) are shown at the top.

We studied the oxidation states of transition-metal elements by using x-ray absorption near-edge spectroscopy (XANES), and the results in Fig. 4A indicate that the involved transition-metal elements are unanimously divalent. In addition, no characteristic increase in Fe pre-edge intensity owing to the deviation from a centrosymmetric ligand field (i.e., *T*_d_) can be found, meaning that the absence of tetrahedrally coordinated Fe(III) in an inverse spinel structure (*63*). Note that pure wüstite FeO is barely accessible due to the aforementioned instability under ambient conditions. Therefore, we can understand a minor shift of Fe *K*-edge for FeO reference material toward higher absorption energy as a result of the oxidation of some Fe(II) to Fe(III). Further, we used Mössbauer spectroscopy to verify the oxidation state of Fe in FeO-HEO.

Figure 4B exhibits a somewhat asymmetric Mössbauer spectrum, indicating a majority of high-spin Fe(II) with the characteristic large isomer shift of about 1 mm/s. To fit the data satisfactorily, we need at least three doublet components [Fe(II)_A,B,C_] with isomer shift around 1 mm/s and a further broad doublet (Fe_D_) with small quadrupole splitting at 0.3 mm/s. We observe the same modeling complexity reported in various stoichiometries of FeO wüstite (*64*). Note that the spectral decomposition is not unique but is the simplest possible that matches the data reasonably well. Fits aiming to stabilize Fe_D_ as a typical Fe(III) fit component with δ ~ 0.3 to 0.4 mm/s systematically revert to the solution presented here. The isomer shifts observed for the three Fe(II) components are similar to those observed for the Fe(II) component in wüstite (FeO*_x_*, 0.95 < *x* < 0.981). However, the quadrupole splitting is considerably larger. Since NPD data suggest no departure from average cubic site symmetry for Fe, this large quadrupole suggests either substantial local distortions or, more likely, a large valence contribution to the quadrupole splitting. A similar observation was reported for FeNCN (*65*), where carbodiimide acts as a rigid linear oxygen analog. Future work is needed to assess whether iron carries an orbital magnetic moment in this FeO-HEO.

The overall spectrum and quadrupole splitting bear more resemblance to those reported for Fe_1-*x*_Mg*_x_*O (keeping in mind that ∆*E*_Q_ = 2ɛ) (*66*). The Fe_D_ component does not match high-spin Fe(III) and Fe^0^ identified in (*64*), but is near in isomer shift to the singlet component identified in mangano-wüstite (δ = 0.22 mm/s). For instance, Gohy *et al.* (*67*) reported that mangano-wüstite also exhibits a shoulder on the left side of the Mössbauer spectrum. A clear identification of the nature of this component is elusive at this point. However, the isomer shift can be ascribed to either a low-spin Fe(II) or intermediate spin Fe(III) (*68*). As a point of reference, stoichiometric FeO is metastable and was reported to have δ = 1.11 mm/s with no visible quadrupole splitting (*69*). Table S5 lists the parameters used to fit the Mössbauer spectrum.

### Magnetic properties

The magnetism in (Mg,Co,Ni,Cu,Zn)O has been investigated extensively. This HEO exhibits long-range AFM order despite its extremely disordered chemical composition (*28*, *29*). The magnetic transition temperature, i.e., Néel temperature (*T*_N_), of FeO-HEO is in the range from 113 to 120 K (*28*, *29*). With the incorporation of large spin magnetic ions such as Fe(II) and Mn(II) and the removal of nonmagnetic ion like Zn(II), the magnetism in (Mg,Mn,Fe,Co,Ni)O is expected to become more robust and informative for the research of magnetic properties of HEOs.

**Fig. 5.**
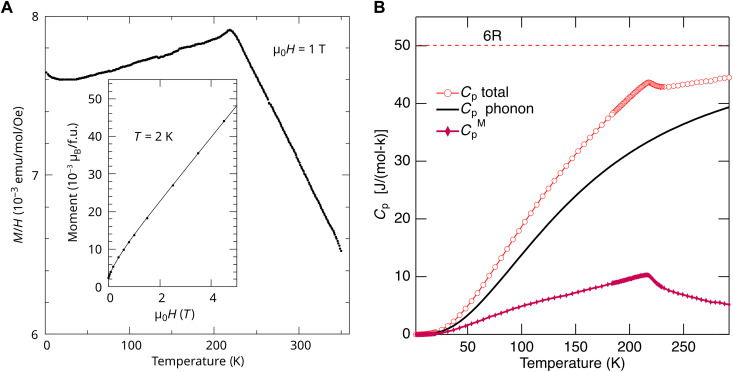
Magnetic properties measurements. (**A**) Temperature dependence of magnetization, *M*/*H*, measured in a 1-T applied field. Inset: Field dependence of magnetization, *M*(*H*), in μ_B_ per formula unit, obtained at 2 K. (**B**) Specific heat for FeO-HEO, *C*_p_ total. The estimated phonon contribution, *C*_p_ phonon, is based on inelastic neutron scattering and is the same as used in (*70*) for (Mg,Co,Ni,Zn,Cu)O. The difference yields the magnetic specific heat, *C*_p_^M^. Dotted line (6R) represents the maximal specific heat.

Figure 5A displays the temperature-dependent magnetization (*M*/*H*) of FeO-HEO measured with a magnetic field of 1 T. The presence of an AFM transition can be observed at *T*_N_ ~ 218 K. The susceptibility with 0.1-T applied field is twice that measured in 1 T (fig. S9). Furthermore, the magnetization data (inset to Fig. 5A) indicate the presence of residual magnetization at zero field. Along with the *M*(*H*) curves in fig. S9, these observations suggest the presence of a small ferromagnetic impurity in the sample, of which the Curie temperature (*T*_C_) is higher than 350 K. Several binary transition-metal oxide phases could be the origin of this impurity. It should be noted that the impurity level in the FeO-HEO is below the detection limit for XRD and NPD. A Curie-Weiss fit to the data is inconclusive, presumably because much higher temperatures are needed to overcome short-range order.

Specific heat measurement reveals a λ-type anomaly associated with the magnetic phase transition at 218 K, as shown in Fig. 5B. This feature is much more pronounced than that in (Mg,Co,Ni,Cu,Zn)O, exhibiting a quasi-continuous magnetic phase transition (*28*). The transition for the FeO-HEO here is clearer, presumably because the average magnetic moment and/or magnetic exchange interaction are vastly larger. A complete separation of the phonon contribution to the specific heat is difficult. A good approximation can be obtained by using the density of phonon state measurement in (Mg,Co,Ni,Cu,Zn)O (*28*), which is reasonably close in composition and average mass. This phonon contribution (*70*), once subtracted from the total specific heat, yields an estimate for the magnetic specific heat, as shown in Fig. 5B.

Integration of *C*_p_^M^/*T* between 2 and 294 K, where *C*_p_^M^ is the magnetic specific heat, provides a released magnetic entropy, *S*^Mag^, of 10.3(5) J/mol/K. Considering that for a transition of a system of moments *J* from fully ordered to fully disordered, the entropy release is *S*_max_ = *R·*ln(2 · *J* + 1), we estimate that *J* = 1.2(1), just below the expected spin-only *J* for this system of 1.4. The estimated magnetic entropy also reveals noteworthy release below the transition, starting at 80 K, and above the transition (*T*_N_ = 218 K); the latter suggests that short-range magnetic correlations subsist well above *T*_N_. A dedicated future study is needed to determine the magnetic structure and correlations.

### Theoretical analysis of HEOs formation

In our previous work, we predicted the relative stabilities of single-phase HEOs formed by mixing five constituent oxides (*36*). All possible combinations were obtained from selecting five elements from a set of eight divalent 3*d* cations, *A* = [Ca, Co, Cu, Fe, Mg, Mn, Ni, Zn]. The enthalpy and entropy descriptors, which are based on the mixing enthalpies of two-component oxides under ambient oxygen partial pressure, are used to perform the prediction. The results suggest that under ambient oxygen partial pressure, five-component oxides (FCOs) containing Mn and Fe have large enthalpy and entropy descriptors. Hence, these FCOs are less prone to form a phase-pure HEO. The low formation abilities of single-phase FCOs containing Mn and Fe under ambient oxygen partial pressure are directly related to the strong phase stability of Mn_2_O_3_ (Δ*H* ≅ −0.36 eV/A-site) and Fe_2_O_3_ (Δ*H* ≅ −0.98 eV/A-site). In other words, attempts to synthesize FCOs containing Fe (Mn) at ambient oxygen partial pressure would produce mixed-phase oxides having rocksalt and Fe_2_O_3_ (Mn_2_O_3_) phases.

However, the oxygen chemical potential, μ_O_, exerts a profound influence on the phase stability of Mn_2_O_3_ and Fe_2_O_3_ compared to that of MnO and FeO. This μ_O_ can be tuned by controlling experimental variables such as temperature, *T*, and oxygen partial pressure, *P*_O_2__. Recently, we demonstrated that the consideration of *P*_O_2__ in our calculations was necessary for understanding the formability of entropy stabilized pyrochlore structures (*71*). In this work, we estimate the phase stability of Fe- and Mn-containing oxides in relation to their stable forms, as a function of *T* and *P*_O_2__. The formation abilities of FCOs containing Mn and Fe are re-evaluated using MnO and FeO as the reference oxides. These formation abilities will be valid under the experimental conditions where MnO and FeO are more stable than Mn_2_O_3_ and Fe_2_O_3_. We find that the formation abilities of FCOs containing Mn and Fe are markedly different, compliant with the stability of their reference oxides. Therefore, we predict that several different HEOs can potentially be synthesized in pure rocksalt structures under low oxygen partial pressure.

First, we estimate the values of temperature and oxygen partial pressures where MnO and FeO oxides are stable. The enthalpy of reaction for, $A_{2}O_{3}\to 2AO+\frac{1}{2}O_{2}$

$$\text{Δ}H_{A}=2\times E_{\mathrm{DFT}}[AO]+\mu_{O}(T,P_{O_{2}})-E_{\mathrm{DFT}}[A_{2}O_{3}]$$ (1)

where, *A* represents Mn or Fe, *E*_DFT_[*A*O] and *E*_DFT_[*A*_2_O_3_] are the density functional theory (DFT) total energy of *A*O and *A*_2_O_3_ oxides, μ_O_(*T*, *P*_O_2__) is the chemical potential of oxygen, calculated using the following equations.

$$\mu_{O}(T,P_{O_{2}})=\frac{1}{2}\left[\mu_{O_{2}}^{0}+{\tilde{\mu }}_{O_{2}}(T,p^{0})+k_{B}T\cdot \ln\left(\frac{p_{O_{2}}}{p^{0}}\right)\right]$$ (2)

$\mu_{O_{2}}^{0}$ is the oxygen chemical potential at ambient pressure *p*^0^ and room temperature, *T*_RT_, and *k*_B_ is the Boltzmann constant. ${\tilde{\mu }}_{O_{2}}(T,p^{0})$ includes the contributions from rotations and vibrations of the oxygen molecule and the ideal gas entropy at 1 atm, tabulated in (*72*, *73*). These are listed in thermodynamic JANAF tables and allow for the determination of the change in the oxygen chemical potential due to the change in temperature and oxygen partial pressure.

**Fig. 6.**
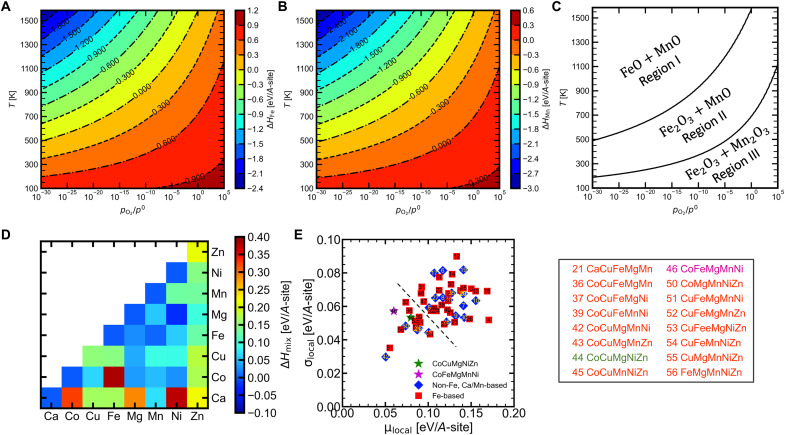
Theoretical guidance on the phase stability with controlled oxygen partial pressure. Δ*H_A_* plotted for (**A**) Fe- and (**B**) Mn-containing oxides calculated from first principle using Eq. 1. The regions labeled with negative and positive values represent stable regions for *A*O and *A*_2_O_3_ phases, respectively. (**C**) Phase diagram showing the stability regions of *A*O and *A*_2_O_3_ phases. (**D**) Mixing enthalpies of two component oxides of all cations in a rocksalt structure under the conditions in region I. (**E**) Comparison of enthalpy and entropy descriptors of all FCOs. The indices of FCOs smaller than the dashed line are provided next to the plot. (Mg,Mn,Fe,Co,Ni)O synthesized in this work is marked as magenta star.

Figure 6 (A and B) presents the calculated reaction enthalpies, Δ*H_A_*, for Fe- and Mn-containing oxides, respectively. The regions labeled with negative values represent the stable regions for *A*O phase, while the positive value–labeled regions are suitable for *A*_2_O_3_ phase formation. Region I shown in Fig. 6C displays region where both FeO and MnO are stable compared to their *A*_2_O_3_ counterparts. We then re-evaluate the enthalpy and entropy descriptors from the mixing enthalpies of two component oxides calculated using MnO and FeO as the reference structures. Hence, the following discussion is valid only in region I.

Figure 6D shows the mixing enthalpies of (*AA*′)O, calculated using Eq. 3

$$\text{Δ}H_{\mathrm{mix}}[(AA^{\prime })O]=E_{\mathrm{DFT}}[(AA^{\prime })O]-\frac{1}{2}E_{\mathrm{DFT}}[AO]-\frac{1}{2}E_{\mathrm{DFT}}[A^{\prime }O]$$ (3)

Δ*H*_mix_[(*AA*′)O] is the mixing enthalpy of (*AA*′)O in rocksalt phase, and *E*_DFT_[(*AA*′)O] is the DFT total energy of structure (*AA*′)O in the rocksalt phase. *E*_DFT_[*A*O] and *E*_DFT_[*A*′O] are the DFT total energies of *A*O and *A*′O structures in their ground structures. Note that in region I (Fig. 6C), both FeO and MnO are stable in rocksalt structure. In addition, while CaO, CoO, MgO, and NiO are stable in rocksalt structure, CuO and ZnO are stable in tenorite and wurtzite structure, respectively (*1*, *36*). The decrease in the mixing enthalpies due to the change in the stability from Mn_2_O_3_ (Fe_2_O_3_) to MnO (FeO) is shown in fig. S10.

Figure 6E exhibits the comparison of enthalpy and entropy descriptors, μ_local_ and σ_local_, respectively, for all 56 combinations of FCOs. The values of μ_local_ and σ_local_ are calculated using the equation 3 (a to d) given in our previous work (*36*). The data points of Fe- and Ca/Mn-containing FCOs are color coded by red squares and blue diamonds, respectively. Furthermore, each data point is annotated with an index that relates to an FCO composition, given in table S6. We find that the range of μ_local_ and σ_local_ for all FCOs is between 0.05 to 0.18 and 0.03 to 0.09 eV, respectively. Compared to the values of μ_local_ and σ_local_ of Fe (0.26 to 0.42 and 0.17 to 0.26 eV)– and Mn/Ca (0.11 to 0.22 and 0.06 to 0.10 eV)–containing oxides in figure 4 of our previous work (*36*), we find that the positions of the FCOs containing Fe have moved to lower values along both μ_local_ and σ_local_ directions. This is owing to the marked decrease in the mixing enthalpies of Mn (Fe)–containing two-component rocksalt oxides, calculated relative to the ground phase structures Mn_2_O_3_ (Fe_2_O_3_) and MnO (FeO), which are stable in region III (region II) and region I, respectively.

The dashed line in Fig. 6E separates the two groups of FCOs, where the group with lower values of μ_local_ and σ_local_ have a higher propensity to form a single-phase HEO. The indices and the composition details are given next to the plot. The FCO-44 combination CoCuMgNiZn, marked as green star, was one of the first HEOs, synthesized by Rost *et al.* (*1*). We further predict that, excluding FCO-44, 15 other FCOs are likely to form HEOs. FCO-46 (i.e., MgMnFeCoNi) synthesized in this work is predicted to have a high propensity to form a single-phase HEO, but under the conditions falling in region I. Furthermore, all other 14 FCOs shown beside Fig. 6E either contain Cu or Zn, which is consistent with our previous finding that when Cu or Zn is present in FCO, the structure may contain tenorite and wurtzite phases, respectively (*36*).

To assess the reliability of the calculation, we calculated the approximate oxygen partial pressure of our annealing system. In the case of no oxygen escape and a complete conversion of MnO_2_ into Mn_3_O_4_, we calculate a maximum *p*_O_2__/*p*^0^ of about 2 × 10^−4^ under our experimental conditions, which is located in region I. One should note that the real value should be lower than this.

## DISCUSSION

The rocksalt HEOs have long been known for their extreme chemical disorder accommodated in single crystal structures. In this work, we report a rocksalt HEO (Mg,Mn,Fe,Co,Ni)O, or preferably, FeO-HEO, containing divalent Fe and Mn. This FeO-HEO in particulate form was prepared by our recently developed bottom-up synthesis strategy. In this rocksalt structure of FeO-HEO, different metal ions reside randomly at cation sites, while the oxygen sublattice works as a buffer layer to accommodate the difference in cationic size. We further validate that this high-entropy configuration can stabilize transition-metal ions like Fe(II) in their oxides, which are notorious for easily oxidizing in ambient environments. In tandem with XANES, we verify the valence state of Fe by using Mössbauer spectroscopy. Both techniques suggest a majority (possibly up to 100%) of divalent Fe in the FeO-HEO. Furthermore, a highly random and homogeneous distribution of both magnetic and nonmagnetic metal ions in a rocksalt structure contributes to a long-range AFM order in this FeO-HEO with a magnetic transition temperature of approximately 218 K. The theoretical analysis predicts that a variety of HEOs can potentially be synthesized in phase-pure rocksalt structures under low oxygen partial pressure. Last but not least, the results of this work pave the way for a deeper understanding of Mn- and Fe-containing rocksalt HEOs.

## MATERIALS AND METHODS

### Materials

Ascorbic acid (C_6_H_8_O_6_), ethylene glycol (C_2_H_6_O_2_), magnesium chloride anhydrous (MgCl_2_), manganese(II) chloride tetrahydrate (MnCl_2_·4H_2_O), iron(II) chloride anhydrous (FeCl_2_), cobalt(II) chloride hexahydrate (CoCl_2_·6H_2_O), nickel(II) chloride hexahydrate (NiCl_2_·6H_2_O), ammonium oxalate monohydrate [(NH_4_)_2_C_2_O_4_·H_2_O], and manganese dioxide (MnO_2_) were all obtained from Sigma-Aldrich. Deionized ultrapure water with a resistivity of 18.2 megohm⋅cm was used for all experiments.

### Synthesis of oxalate precursor

In a typical procedure, 0.1 mmol of ascorbic acid (C_6_H_8_O_6_) was first dissolved in a mixed solution of 15 ml of deionized H_2_O and 15 ml of ethylene glycol. Then, 1.1 mmol of MgCl_2_ (98%), 1 mmol of MnCl_2_·4H_2_O (98%), 1 mmol of FeCl_2_ (98%), 1 mmol of CoCl_2_·6H_2_O (99%), and 1 mmol of NiCl_2_·6H_2_O (100%) were dissolved into the above solution in a round-bottom flask. In another mixed solution of deionized H_2_O and ethylene glycol (15 ml + 15 ml), 5.1 mmol of ammonium oxalate monohydrate (NH_4_)_2_C_2_O_4_·H_2_O was slowly dissolved at 50°C. The solution of ammonium oxalate was then rapidly poured into the solution of chlorides at 50°C. Oxalate precursors can be obtained after a 6-hour reaction. The product was washed and separated via centrifugation for a couple of times, followed by drying the precursor at 50°C overnight. The detailed synthesis method can be found in the Supplementary Materials, and a future publication will be dedicated to elaborating the mechanism of this synthesis strategy.

### Annealing of oxalate precursor

To avoid the oxidation of Fe(II) and Mn(II), we conducted the annealing in a tube furnace filled with argon. In a typical process, the precursor was calcined using a tube furnace at 1000°C for 6 hours. A measured 300 mg of the precursor was placed in a quartz pan within the boat, and 90 mg of MnO_2_ as an oxygen generator was situated next to the quartz pan. The FeO-HEO oxide can be obtained after cooling down the sample naturally, and the as-obtained dark brown powders are proved to be stable in ambient environment. The flow rate of argon gas was set at 50 ml/min during the entire annealing process.

### Characterizations

The chemical composition of the oxalate precursor was analyzed by using inductively coupled plasma optical emission spectrometry. Analysis was undertaken using corresponding calibration solutions. All ex situ XRD patterns were acquired on a Rigaku Ultima IV laboratory diffractometer with Cu-Kα radiation in plate mode. The in situ XRD measurements were performed on the diffractometer with a high-temperature attachment. Quasi in situ XPS measurements were carried out on Thermo Escalab 250 XI with a monochromatic Al-Kα source. A Mössbauer spectrum of (Mg,Mn,Fe,Co,Ni)O powder was recorded at room temperature in the ±4 mm/s velocity range using a Wissel CMCA-500 multichannel analyzer. Magnetic properties were measured on a piece of pressed (Mg,Mn,Fe,Co,Ni)O pellet with a Quantum Design (QD) Magnetic Property Measurement System in the temperature range 2.0 < *T*/K < 350 K and in applied magnetic fields 1 T, and at 2 K in variable field between 0 and 6 T. Zero-field cooled and field cooled data were collected between 2 and 320 K under an applied field of 0.1 T. The specific heat data were collected on a 16.6-mg pellet of (Mg,Mn,Fe,Co,Ni)O between 2 and 350 K using a 9-T QD Physical Property Measurement System in a zero applied magnetic field. SEM images and atomic-scale characterizations of FeO-HEO oxide were performed on a ZEISS Gemini 500 and an aberration-corrected STEM (FEI Titan Cubed Themis G2 300, FEI), respectively. Further characterization details are reported in the Supplementary Materials.

### Neutron diffraction and x-ray absorption spectroscopy

Neutron diffraction data were obtained on 2 g of powder at 300 K with a wavelength band centered at 0.8 Å (*d*-spacing coverage 0.1 to 8 Å) using the time-of-flight powder neutron diffractometer POWGEN at the Spallation Neutron Source (SNS) at Oak Ridge National Laboratory (ORNL), USA. The diffraction data of the sample under long-term exposure to air were collected on the high-resolution powder diffractometer Echidna at the Australian Centre for Neutron Scattering. The measurement was performed in an angular range of 4° to 164° with a neutron wavelength of around 1.2992(1) Å, which was determined using NIST Standard Reference Material 660b La^11^B_6_. Rietveld refinements were performed using the FULLPROF software (*74*). All refinement results are reported in table S7 (Supplementary Materials).

Both XANES and EXAFS measurements were conducted at the XAS beamline at the Australian Synchrotron by applying a set of Si (111) monochromator crystals. Spectra were recorded at the Mn *K*-edge (6539 keV), Fe *K*-edge (7112 keV), Co *K*-edge (7709 keV), and Ni *K*-edge (8333 keV) in transmission mode. Pre-edge spectra were collected via an energy step size of 5 eV, while for the XANES region, it was 0.25 eV. A dwell time of 2 s was applied for both pre-edge and XANES data collection. EXAFS spectra were acquired with a maximum *K* value of 12 for Mn, Fe, Co, and 14 for Ni. The *k*-step size was 0.035 Å^−1^, with a maximum dwell time of 4 s at each step. The XAS data reduction and processing were performed using the ATHENA software package through standard methods, and curve fitting was performed with Artemis (*75*).

### Computational methods

All DFT calculations used in this work are same as that were used in our previous work (*36*). These were carried out using the plane wave–based Vienna Ab-initio simulation Package VASP (*76*, *77*) version 5.4.4, within the generalized gradient approximation (GGA) using the Perdew-Burke-Ernzerhof for solids (PBEsol) exchange-correlation functional (*78*). The energy cutoff for the plane-wave basis set was 800 eV, using projected augmented wave potentials (*79*, *80*). An 888 *k*-point mesh was used for sampling the Brillouin zone for a two-atom unit cell and scaled linearly with the number of atoms present in the unit cell. The bulk geometry was optimized with a force convergence criterion of 1 meV/Å, and the individual components of the stress tensor were converged to ≤0.1 kB. Magnetism of Co, Cu, Fe, Mn, and Ni oxides was treated with the PBEsol collinear spin density approximation in the GGA with an onsite Hubbard *U* (GGA + *U*) scheme (*81*). An on-site coulomb parameter *U* = 6 eV was applied for all cations to account for the increased coulomb repulsion between the semi-filled 3*d* states.

## Supplementary Materials

### This PDF file includes:

Supplementary Text Figs. S1 to S10 Tables S1 to S7 References
