# Supplementary material for: (Mg,Mn,Fe,Co,Ni)O: A rocksalt high-entropy oxide containing divalent Mn and Fe
Source: Sci Adv. 2023 Sep 20;9(38):eadi8809. doi: 10.1126/sciadv.adi8809 (PMC10511202; doi:10.1126/sciadv.adi8809)
Supplement: Supplementary file 1 — Supplementary Text Figs. S1 to S10 Tables S1 to S7 References [file sciadv.adi8809_sm.pdf]

Science Advances
AAAS

Supplementary Materials for

(Mg,Mn,Fe,Co,Ni)O: A rocksalt high-entropy oxide containing divalent Mn and Fe

Yuguang Pu *et al.*

Corresponding author: Raphael P. Hermann, hermannrp@ornl.gov; Peng Cao, p.cao@auckland.ac.nz

Sci. Adv. **9**, eadi8809 (2023)
DOI: 10.1126/sciadv.adi8809

This PDF file includes:

Supplementary Text
Figs. S1 to S10
Tables S1 to S7
References

Supplementary Text

Experimental details

Synthesis of oxalate precursor

The synthesis of oxalate precursor involves a co-precipitation of different metal ions into fine particles. Some involved divalent cations (i.e., Fe^{2+} and Mn^{2+}) are vulnerable to oxygen, meaning that they are prone to be oxidized to their higher valence states. Therefore, the fabrication of the precursor was undertaken in an inert atmosphere using Schlenk lines, guaranteeing a pure Ar atmosphere during the wet synthesis. In a typical procedure, 0.1 mmol ascorbic acid ($C_6H_8O_6$) was first dissolved in a mixed solution of 15 ml deionized H_2O and 15 ml ethylene glycol. Ascorbic acid, which is a reducing agent, can effectively prevent the oxidation of Fe^{2+} and Mn^{2+} in the aqueous solution. Then, 1.1 mmol $MgCl_2$ (98%), 1 mmol $MnCl_2 \cdot 4H_2O$ (98%), 1 mmol $FeCl_2$ (98%), 1 mmol $CoCl_2 \cdot 6H_2O$ (99%), and 1 mmol $NiCl_2 \cdot 6H_2O$ (100%) were dissolved into the above solution in a round bottom flask. After all metal chlorides were added to the solution, the flask was swiftly connected to the Schlenk line and purged with argon gas for three times. This solution of metal chlorides was warmed up to 50 °C under stirring. In another mixed solution of deionized H_2O and ethylene glycol (15 ml+15 ml), 5.1 mmol ammonium oxalate monohydrate $(NH_4)_2C_2O_4 \cdot H_2O$ was slowly dissolved at 50 °C. This solution was then deoxygenated using the Schlenk line before being injected into the solution of chlorides under vigorous stirring. After a reaction for 6 hours, the oxalate precursor was washed and separated via centrifugation for a couple of times, followed by drying the precursor at 50 °C overnight.

Annealing of oxalate precursor

A single-phase FeO-HEO can be obtained by annealing the precursor at a high temperature. To avoid the oxidation of Fe^{2+} and Mn^{2+}, we conducted the annealing in a tube furnace filled with argon. The tube atmosphere was purged with pure Ar for three times before the annealing. A certain amount of oxygen was introduced to the system to effectively counteract the reductive environment created by the thermal decomposition of oxalate in inert atmosphere. Specifically, the precursor was calcined using a tube furnace at 1000 °C for 6 h with a ramp rate of 10 °C/min in an Ar atmosphere within a partially lidded corundum ceramic boat. A measured 300 mg of precursor was placed in a quartz pan within the boat, and 90 mg of MnO_2 as an oxygen generator was situated next to the quartz pan. The use of quartz pan allowed the MnO_2 to be situated close to the precursor sample, while preventing contact and contamination. The FeO-HEO can be obtained after cooling down the sample naturally to the room temperature, and the as-obtained dark brown powders are proved to be stable in ambient environment, as shown in Figure S5, the phase purity shows no change after exposing the sample to air for months.

Characterizations

The chemical compositions of the oxalate precursor were analyzed by using inductively coupled plasma optical emission spectroscopy (ICP-OES). 10 mg of oxalate sample was dissolved in 20 ml 0.1 M nitric acid at the room temperature. Analysis was undertaken using corresponding calibration solutions.

All ex-situ X-ray diffraction patterns were acquired on a Rigaku Ultima IV laboratory diffractometer with Cu Kα radiation in plate mode. The in-situ XRD measurements were performed on the diffractometer with a high-temperature attachment. The sample was heated up to different stages with a ramp rate of 10 °C/min, and a dwelling time of 10 min was adopted before each scan.

Quasi in-situ XPS measurements were carried out on Thermo Escalab 250 XI with a monochromatic Al-Kα source. The sample was annealed at different temperatures in an isolated

oven and was transferred into the ultra-high vacuum chamber without exposure to air. All spectra were initially calibrated with the C 1s peak of adventitious carbon at 284.8 eV before any fitting.
A Mössbauer spectrum of 44 mg/cm^2 of (Mg,Mn,Fe,Co,Ni)O powder was recorded at room temperature in the ± 4 mm/s velocity range with a krypton gas proportional counter recording the 1.8 keV Kr K-α escape peak and 14.41 keV resonant photons using a Wissel CMCA-500 multichannel analyzer. The spectrometer was calibrated at room temperature using α-iron, which serves as isomer shift reference.

Magnetic properties were measured on a piece of pressed (MgMnFeCoNi)O pellet with a Quantum Design (QD) Magnetic Property Measurement System in the temperature range 2.0 < T/K <350 K and in applied magnetic fields 1 T and at 2 K in variable field between 0 and 6 T. Zero-field cooled and field cooled data were collected between 2 and 320 K under with an applied field of 0.1 T. The specific heat data were collected on a 16.6 mg pellet of (MgMnFeCoNi)O between 2 and 350 K using a 9T QD Physical Property Measurement System (PPMS) in zero applied magnetic field.

SEM images were taken on a field-emission scanning electron microscope (ZEISS Gemini 500) operating at 30 kV. The atomic-scale characterizations of individual FeO-HEO particle were conducted on an aberration-corrected STEM (FEI Titan Cubed Themis G2 300, FEI) at an accelerating voltage of 300 kV with a convergence semi-angle of 25 mrad.

Wet-chemistry synthesis of oxalate precursor

The synthesis strategy of a precursor using oxalate anion as the ‘bridging’ ligand will be elucidated specifically in another work published elsewhere. Critical stability constants of involved metal-oxalate complexes are listed in Table S1. The complexes formed between oxalate and Ni^{2+} are the most stable among these five cations, followed by the Co^{2+} containing complexes. The stabilities of Fe- and Mg-oxalate complexes are relatively close and slightly lower than others. The chemical compositions determined by ICP-OES are shown as the inset in Figure S1A. The molar fraction of each metal component is close to 20%, while that of Mg is lower than others reaching a percentage of 18%. This diminution in Mg content may be ascribed to the slight dissolution of Mg oxalate during washing. Figure S1 show the SEM images of oxalate precursor. The images at low magnification (Figure S1B) and high magnification (Figure S1C) indicate that the as-synthesized oval precursor is about 3 to 4 μm in length and has a maximum width of 800 nm around the waist.

Annealing of oxalate precursor

As shown in Figure S2, direct annealing of the oxalate precursor in air results in the formation of a spinel product, which is due to the further oxidization of Co^{2+} and Fe^{2+} to their higher valence states. Therefore, to synthesize the single-phase rocksalt FeO-HEO, in which different metal ions co-occupy the cation sites, all cations are supposed to be bivalent in this structure. The precursor was then annealed in an argon atmosphere with a certain amount of oxygen generator (that is, MnO_2 in our case) to obtain a rocksalt FeO-HEO.

However, the annealing process in pure Ar leads to a mixture of wüstite (FeO) and Ni alloy (Figure S3A). The presence of metallic phase can be ascribed to the generation of reductive by-products (i.e., carbon and carbon monoxide) during the heat treatment (*42*). In this regard, MnO_2 is introduced into the annealing process as an oxygen generator to offset the reductive environment. MnO_2 decomposes progressively at high temperatures and releases a small amount of O_2, either neutralizing the reductive substances or slightly re-oxidizing the as-formed metallic products. MnO_2 undergoes a thermal decomposition as follows(*43*):

$$(400°C\sim800\ °C)\quad 2MnO_2 = Mn_2O_3 + 1/2O_2 \qquad (1)$$

(Above 800°C) $3Mn_2O_3 = 2Mn_3O_4 + 1/2O_2$ (2)

Figures S3 B-D show the XRD patterns of the samples annealed with the addition of MnO_2 as an external oxygen source. An excess of MnO_2 produces a spinel product (Figure S3B). This spinel phase forms because Fe^{2+}, Mn^{2+}, and Co^{2+} cations can be possibly oxidized to Fe^{3+}, Mn^{3+}, and Co^{3+} by extra oxygen generated from MnO_2. A variety of normal and inverse spinel oxides are likely to form during this process.(*82*) Hence, it is necessary to control the amount of MnO_2 properly.

Figure S3C exhibits the co-existence of spinel and metallic phases in the annealed product with the controlled addition of MnO_2. Figure S4 illustrates an insufficient annealing process schematically. Specifically, due to a limited oxygen diffusion rate within the fixed bed, insufficient annealing results in an overoxidized top layer and an under oxidized bottom layer. Such a problem can be addressed by prolonging the annealing time. As shown in Figure S3D, compared with the product in Figure S3C, when the precursor was annealed at the same temperature with the same amount of MnO_2, an annealing time more than 5 hours results in well-crystallized single-phase FeO-HEO.

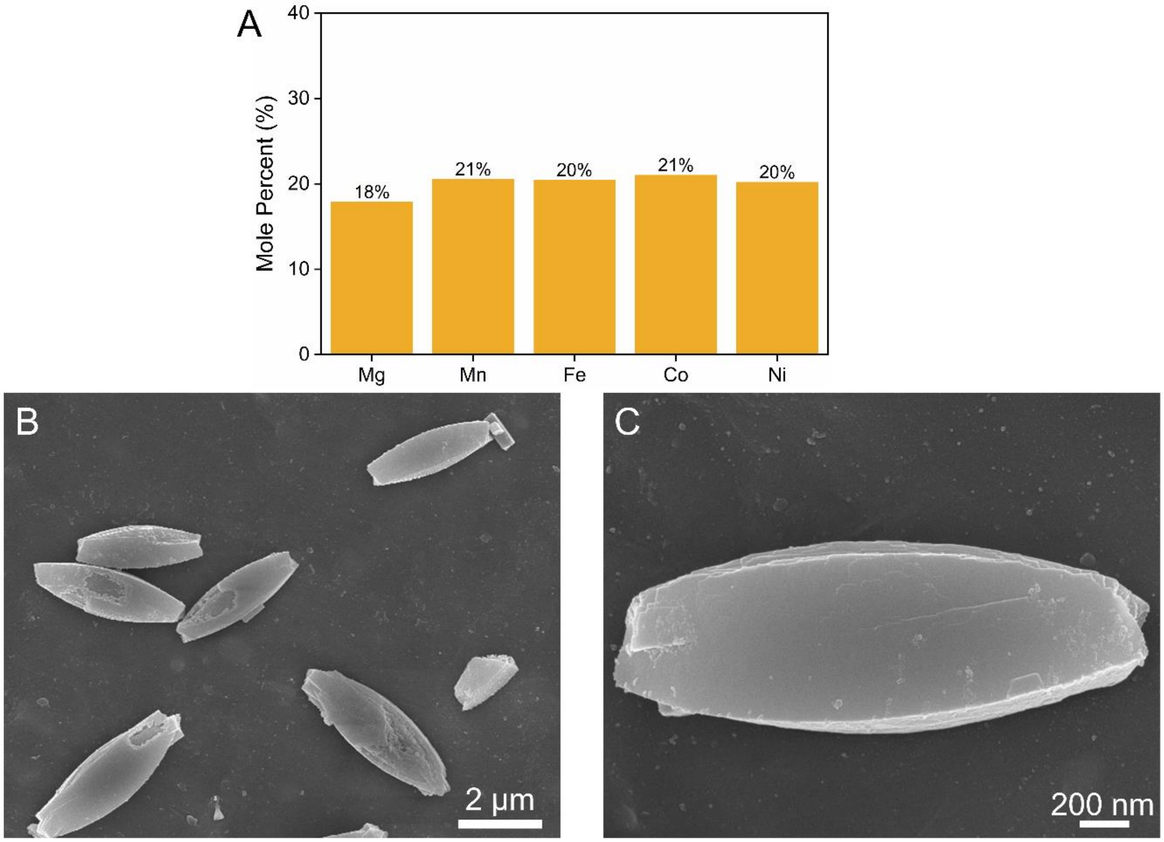

Fig. S1.

Characterizations of the oxalate precursor. ICP-OES results (**A**) and SEM images of oxalate precursor at low magnification (**B**) and high magnification (**C**).

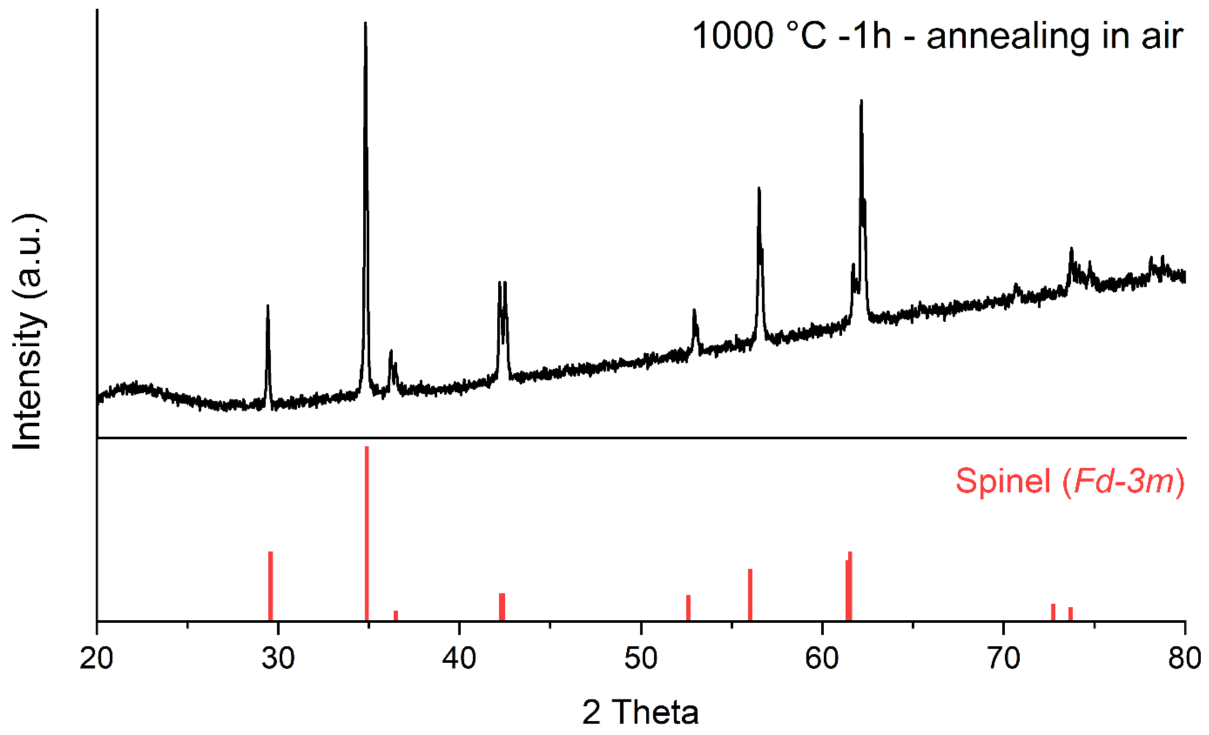

Fig. S2.

XRD pattern of the sample annealed at 1000 °C for 1 h in air.

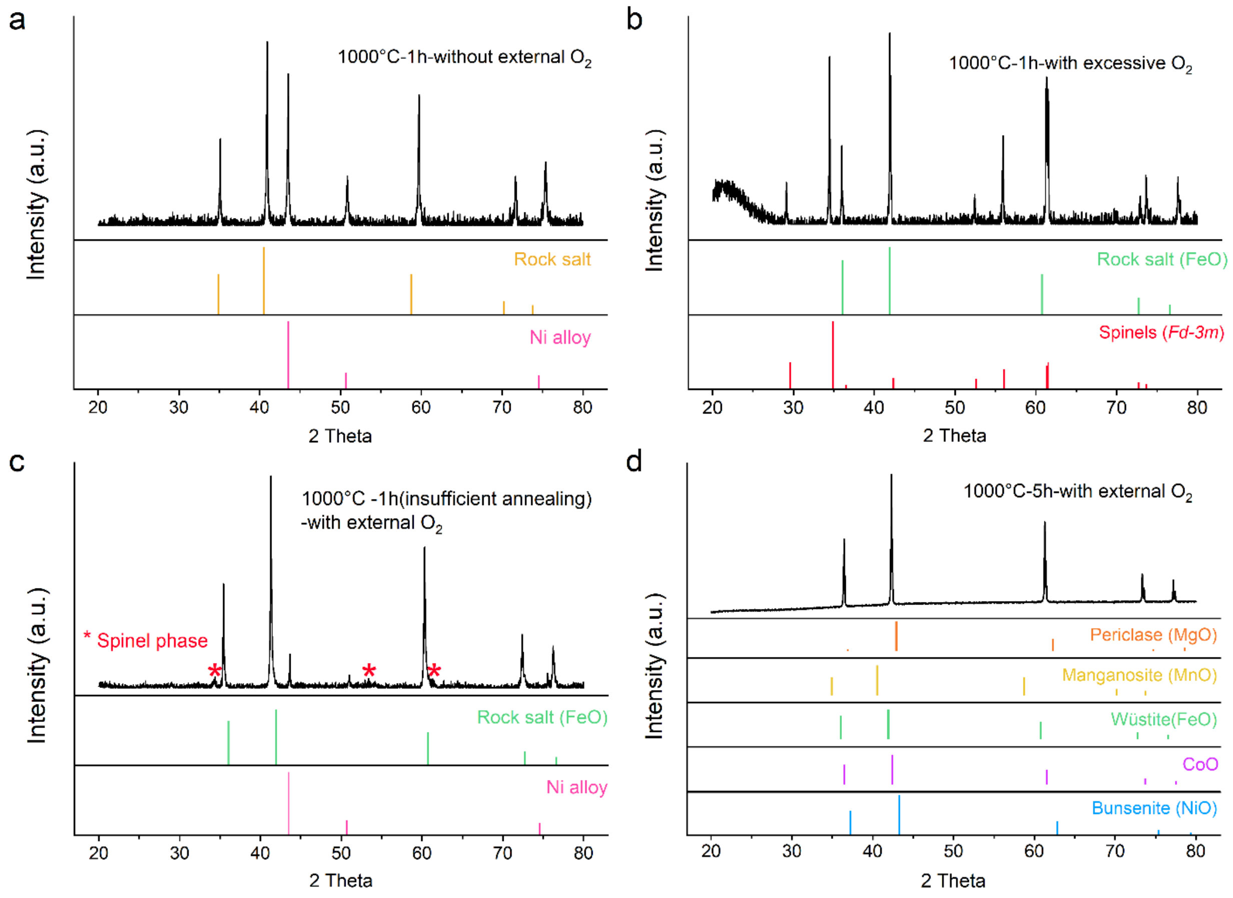

Fig. S3.

XRD patterns of samples annealed under different conditions. A) the sample annealed at 1000 °C for 1 h in Ar atmosphere. **B**) the sample annealed at 1000 °C for 1 h in Ar atmosphere with the presence of an excess of O_2. **C**) the sample annealed at 1000 °C for 1 h in Ar with an insufficient O_2 supply. **D**) the sample annealed at 1000 °C for 5 h in Ar atmosphere with a suitable amount of O_2.

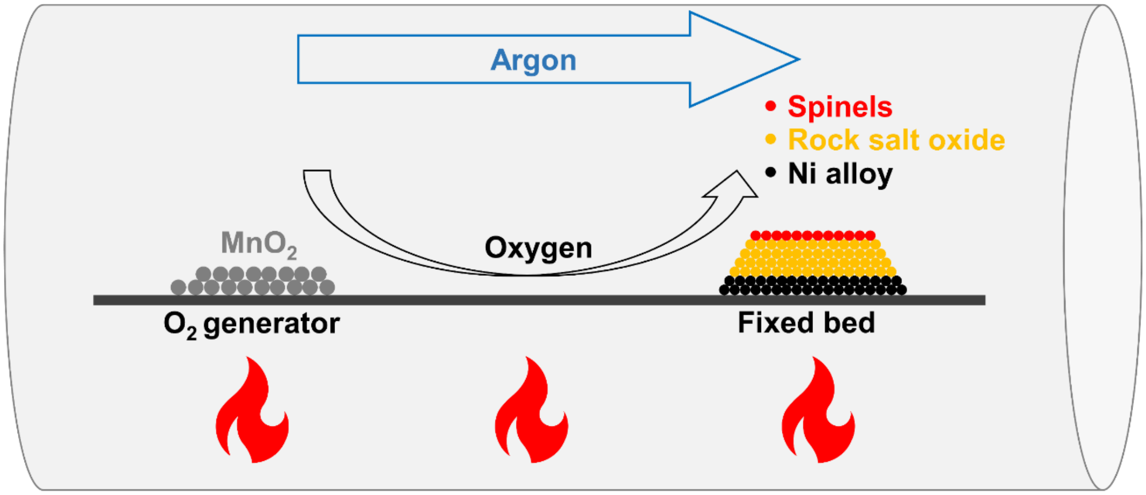

Fig. S4.

Schematic illustration of an insufficiently annealed fixed bed in the tube furnace.

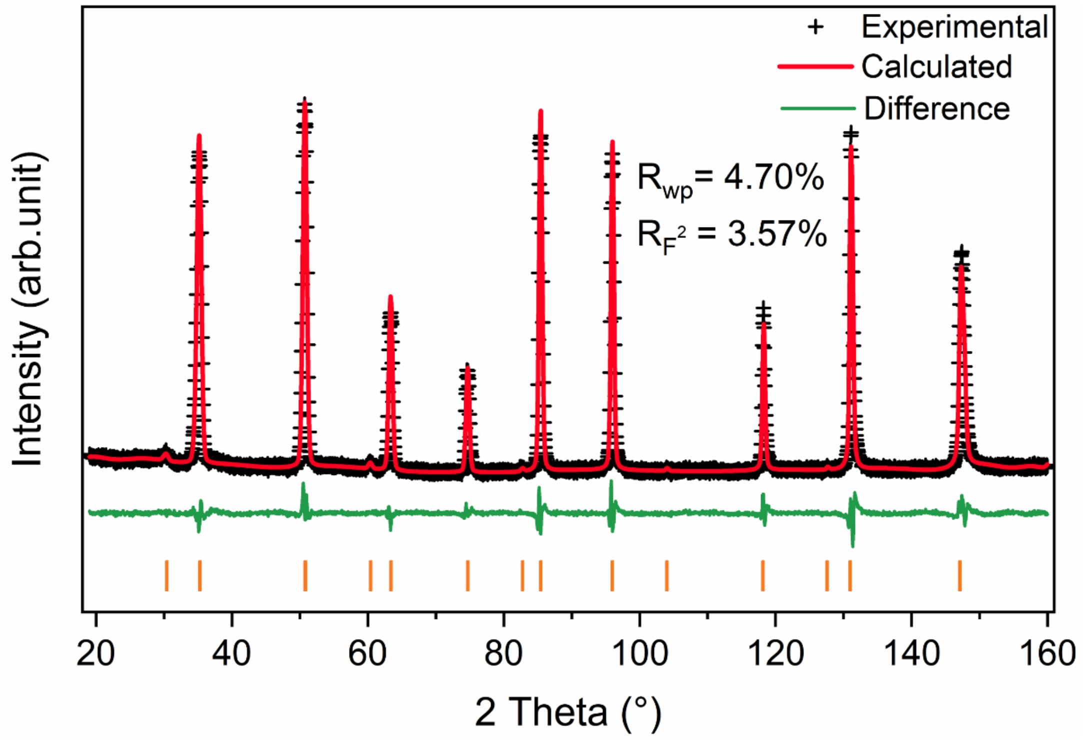

Fig. S5.

Rietveld refinement profile using high-resolution NPD data (Echidna, ACNS, ANSTO) collected at a wavelength of 1.2992(1) Å. Figure S5 shows the neutron diffraction data collected on the sample that has been exposed to air for 3 months. Figures of merit for the refinement were the weighted profile R factor R_{wp} = 4.7% and the Bragg R factor $R_F{}^2$ = 3.57%. The latter is an indicator of how well the phase is being described by the data, and a value of 3.57% implies a reliable phase model (*83*). The refined crystal structure based on neutron diffraction further corroborates that this FeO-HEO sample has a single rocksalt phase. Refinement results are reported in Table S7. These refinement results suggest a centrosymmetric *fcc* lattice with a unit-cell parameter a=b=c=4.282 Å, which agrees with the results obtained from XRD refinement and NPD data collected at SNS, ORNL.

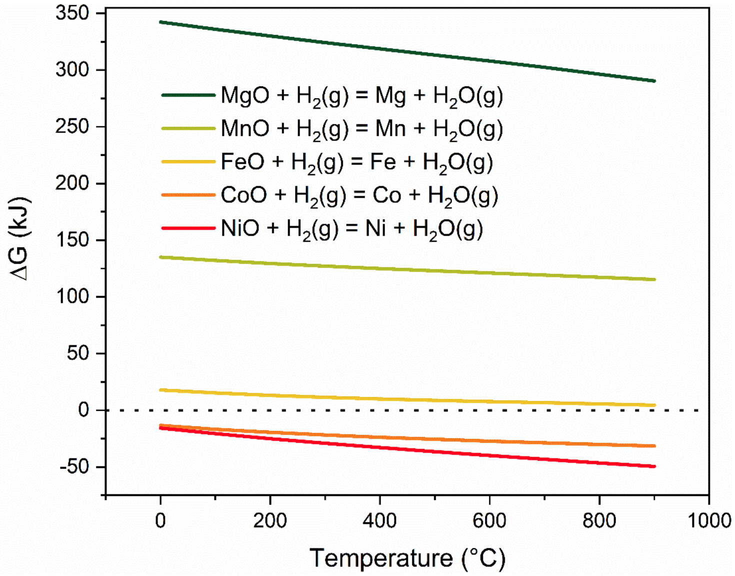

Fig. S6.

Calculated thermodynamic data of the reduction of involved binary oxides. Figure S6 shows the calculated thermodynamic data of the reduction of constituent binary oxides in a 5% H_2 atmosphere. It is apparent that only NiO and CoO among all component oxides can be reduced into their metallic states ($\Delta G < 0$) in a temperature range from 0 °C to 1000 °C.

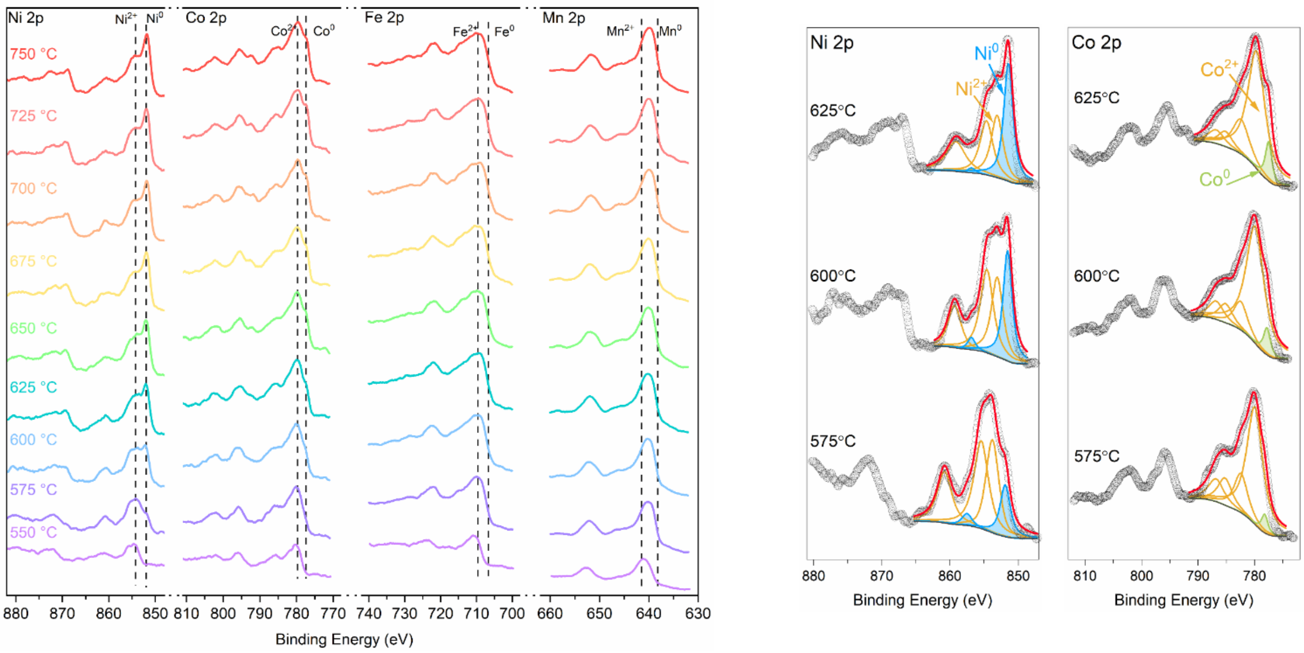

Fig. S7.

Quasi in-situ XPS results of the FeO-HEO reduced at different temperatures. Left) Ni 2p, Co 2p, Fe 2p, and Mn 2p spectra over increased reduction temperature (offset in y for clarity). Right) Fitted Ni 2p and Co 2p spectra collected at 575 °C, 600 °C, and 625 °C, respectively.

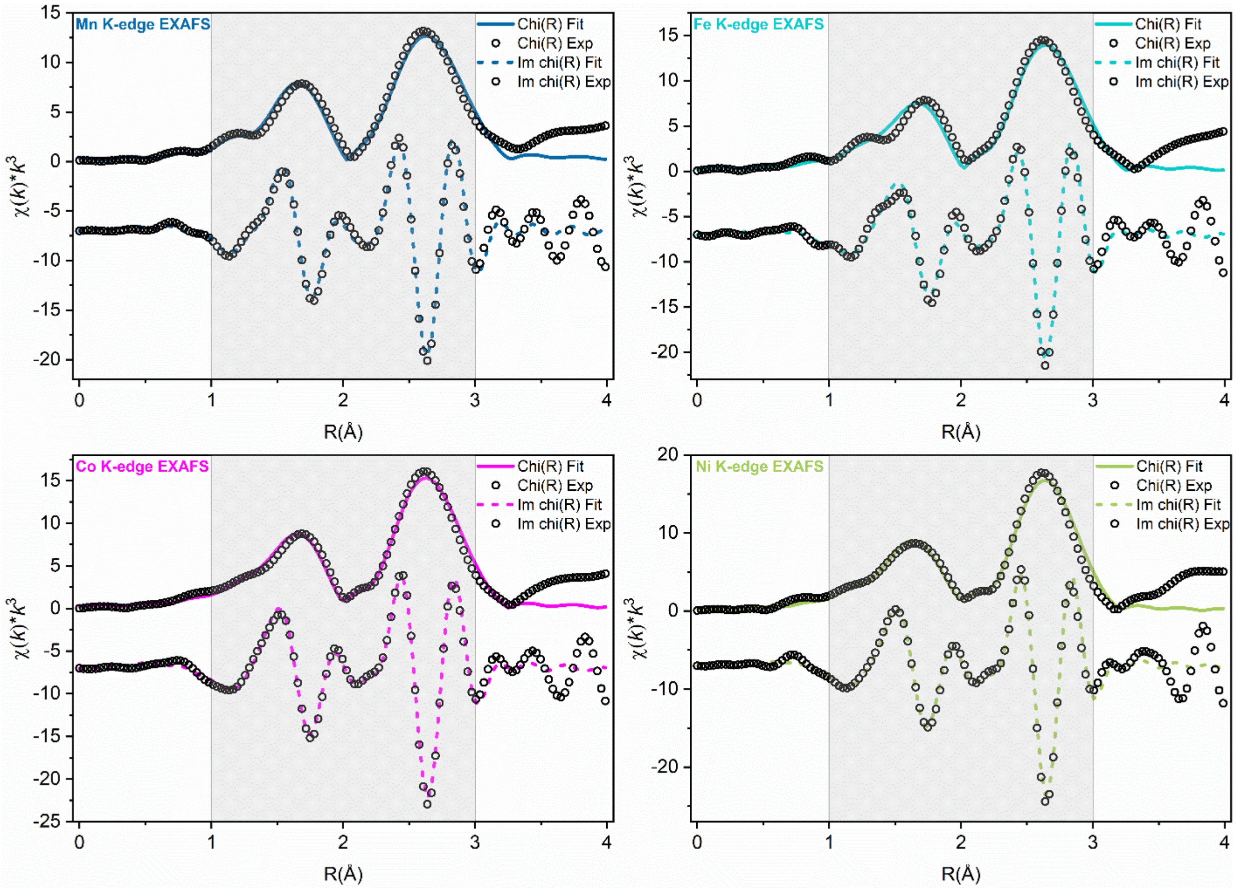

Fig. S8.

Fourier transforms of $\chi(k)\cdot k^3$ oscillations (top scatter plots) for Mn, Fe, Co, and Ni K-edges and respective imaginary parts (bottom scatter plots). The curve fits of chi(R) data (solid) and imaginary parts (dash) are demonstrated as colored lines. The fit range is from 1 to 3 Å consistently in all cases, and fit windows are shaded in grey in each figure.

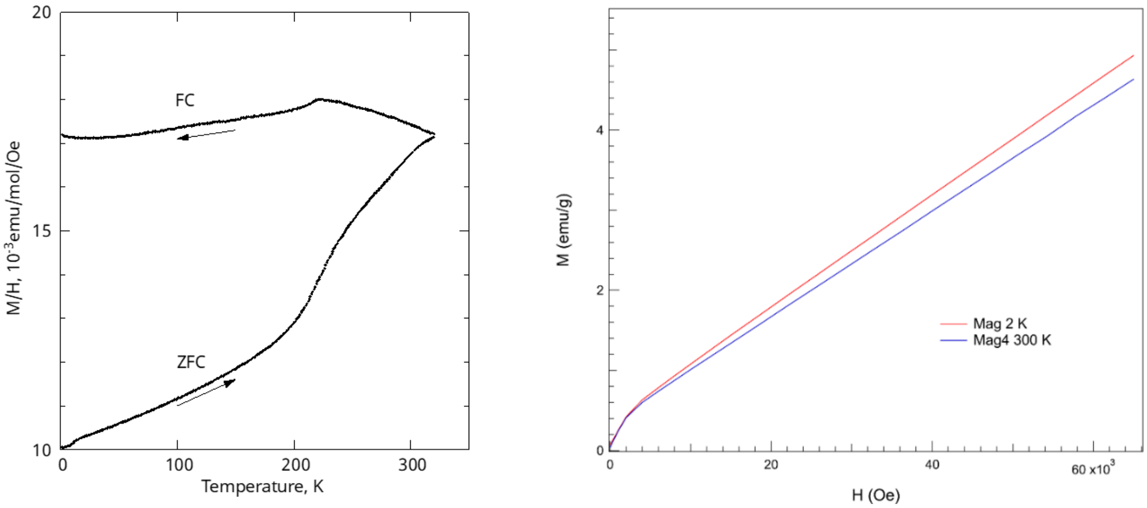

Fig. S9.

Field-cooled (FC) and zero field cooled (ZFC) magnetization data for FeO-HEO (left) and MH curves measured at 2 K and 300 K (right).

An absence of overlap between the FC and ZFC curves indicates the presence of a small amount of ferromagnetic impurity. The similarity in MH curves suggests the retention of this weak ferromagnetism even at room temperature.

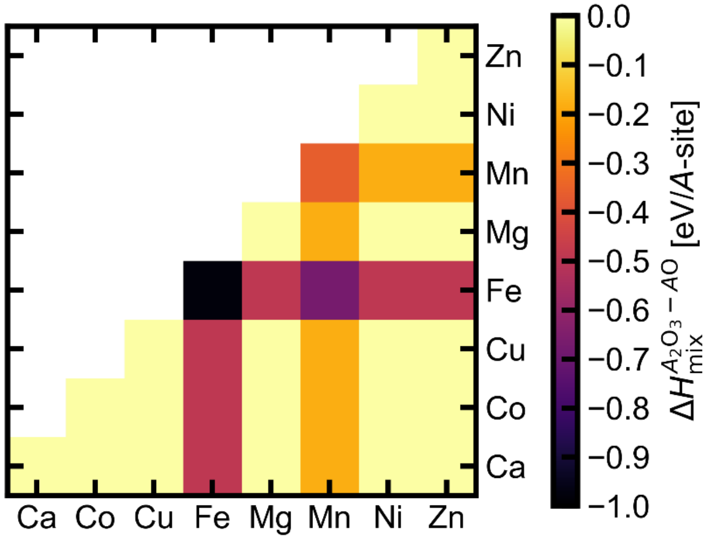

Fig. S10.

The decrease in the mixing enthalpies due to the change in the stability from Mn_2O_3 (Fe_2O_3) to MnO (FeO).

		Log K (25℃, 0)
Mg^{2+}	ML/M.L.	3.43
Mn^{2+}	ML/M.L.	3.950.03
Fe^{2+}	ML/M.L.	3.5
Co^{2+}	ML/M.L.	4.720.08
Ni^{2+}	ML/M.L.	5.16

Table S1.

Critical stability constants of metal-oxalate complexes.(*84*, *85*)

	Mg	Mn	Fe	Co	Ni
X-rays Scattering cross-sections (Cu Ka, cm^2/g)	0.68	1.407	1.542	1.626	1.811
Neutron Scattering cross-sections (10^{-24} cm^2)	3.63	1.75	11.22	0.779	13.3

Table S2.

X-ray and Neutron coherent scattering cross-sections (*49*).

Scattering cross-section (σ) is commonly employed to describe the scattering power of X-rays and neutrons by a certain atom. This scattering cross-section is proportional to the square of scattering length (b), which can be expressed as $\sigma = 4\pi b^2$. Therefore, neutron diffraction is a powerful complementary tool for XRD to characterize the crystal structures and distinguish elements in the proximity.

	B_{iso} (Å^2, metal)	B_{iso} (Å^2, oxygen)	Source
(MgCoNiCuZn)O	0.32	0.45	NPD data
FeO-HEO	0.475(15)	0.612(15)	NPD data
MgO	0.312(7)	0.362(8)	Ref (*45*)
NiO	0.414(8)	0.61(3)	Ref (*45*)
CoO	0.509(6)	0.67(2)	Ref (*45*)
MnO	0.617(5)	0.72(2)	Ref (*45*)
FeO	0.16	-	Ref (*86*)
CuO	Not rocksalt		
ZnO			

Table S3.

Atomic displacement parameters of metals and oxygen in different oxides.

Rietveld refinement thermal factors for FeO are not provided. This B_{iso} value (Fe^{2+}) is calculated from the Mossbauer recoil-free fraction. Ref (*86*) reported an f_{LM} around 0.775 for (Mg,Fe)O, where f_{LM} is the Mossbauer recoil-free fraction and $f_{LM} = e^{(-k^2u^2)}$,where k=2π/λ for iron Mossbauer spectroscopy (E = 14.41keV, λ = 0.8604).

Absorber	Path	N	S_0^2	R/Å	σ^2/Å^2
Mn	Mn-O	6.09	0.83	2.13	0.008(1)
	Mn-Me	12.08		3.03	0.010(1)
Fe	Fe-O	6	0.92	2.12(1)	0.009(1)
	Fe-Me	12		3.03(1)	0.010(1)
Co	Co-O	6.1	1	2.10(1)	0.009(2)
	Co-Me	12		3.02(1)	0.010(1)
Ni	Ni-O	6	0.95	2.09(1)	0.007(1)
	Ni-Me	11.8		3.03(1)	0.009(1)

Table S4.

Fitting parameters for Mn, Fe, Co, and Ni K-edge EXAFS results for the FeO-HEO.

	Area, %	Γ, mm/s	δ, mm/s	ΔE_Q, mm/s
$Fe(II)_A$	41(2)	0.46(1)	1.082(1)	0.85(1)
$Fe(II)_B$	30(1)	0.43(2)	1.083(1)	1.26(1)
$Fe(II)_C$	11(1)	0.36(2)	1.086(3)	1.78(1)
Fe_D	18(2)	0.51(2)	0.140(3)	0.29(1)

Table S5.

Mössbauer spectral parameters obtained from a fit to the room-temperature data. Mössbauer spectrum with four components, where Γ, δ, and ΔE_Q, are the full width at half-maximum, the isomer shift, and the quadrupole splitting, respectively.

**1**	Ca Co Cu Fe Mg	**15**	Ca Co Fe Mn Zn	**29**	Ca Cu Mg Ni Zn	**43**	Co Cu Mg Mn Zn
**2**	Ca Co Cu Fe Mn	**16**	Ca Co Fe Ni Zn	**30**	Ca Cu Mn Ni Zn	**44**	Co Cu Mg Ni Zn
**3**	Ca Co Cu Fe Ni	**17**	Ca Co Mg Mn Ni	**31**	Ca Fe Mg Mn Ni	**45**	Co Cu Mn Ni Zn
**4**	Ca Co Cu Fe Zn	**18**	Ca Co Mg Mn Zn	**32**	Ca Fe Mg Mn Zn	**46**	Co Fe Mg Mn Ni
**5**	Ca Co Cu Mg Mn	**19**	Ca Co Mg Ni Zn	**33**	Ca Fe Mg Ni Zn	**47**	Co Fe Mg Mn Zn
**6**	Ca Co Cu Mg Ni	**20**	Ca Co Mn Ni Zn	**34**	Ca Fe Mn Ni Zn	**48**	Co Fe Mg Ni Zn
**7**	Ca Co Cu Mg Zn	**21**	Ca Cu Fe Mg Mn	**35**	Ca Mg Mn Ni Zn	**49**	Co Fe Mn Ni Zn
**8**	Ca Co Cu Mn Ni	**22**	Ca Cu Fe Mg Ni	**36**	Co Cu Fe Mg Mn	**50**	Co Mg Mn Ni Zn
**9**	Ca Co Cu Mn Zn	**23**	Ca Cu Fe Mg Zn	**37**	Co Cu Fe Mg Ni	**51**	Cu Fe Mg Mn Ni
**10**	Ca Co Cu Ni Zn	**24**	Ca Cu Fe Mn Ni	**38**	Co Cu Fe Mg Zn	**52**	Cu Fe Mg Mn Zn
**11**	Ca Co Fe Mg Mn	**25**	Ca Cu Fe Mn Zn	**39**	Co Cu Fe Mn Ni	**53**	Cu Fe Mg Ni Zn
**12**	Ca Co Fe Mg Ni	**26**	Ca Cu Fe Ni Zn	**40**	Co Cu Fe Mn Zn	**54**	Cu Fe Mn Ni Zn
**13**	Ca Co Fe Mg Zn	**27**	Ca Cu Mg Mn Ni	**41**	Co Cu Fe Ni Zn	**55**	Cu Mg Mn Ni Zn
**14**	Ca Co Fe Mn Ni	**28**	Ca Cu Mg Mn Zn	**42**	Co Cu Mg Mn Ni	**56**	Fe Mg Mn Ni Zn

Table S6.
Index of the FCOs with all possible cation combinations.

Refinement details based on XRD results

Space group *Fm*-3*m*						
a= 4.28478(2) Å, volume = 78.666(3) $Å^3$						
**Atom**	**Wyckoff site**	***x***	***y***	***z***	**B_{iso} ($Å^2$)**	**Site occupancy factor**
Mg	4*a*	0	0	0	0.353(16)	0.1996
Mn	4*a*	0	0	0	0.353(16)	0.2101
Fe	4*a*	0	0	0	0.353(16)	0.1901
Co	4*a*	0	0	0	0.353(16)	0.2101
Ni	4*a*	0	0	0	0.353(16)	0.1901
O	4*b*	0.5	0.5	0.5	0.100(27)	1.0
**R_{wp} 1.612%, GOF 1.385**						

Refinement details based on NPD data collected on Echidna at ACNS

Space group *Fm*-3*m*						
a= 4.2818(1) Å, volume = 78.5022(6) $Å^3$						
**Atom**	**Wyckoff site**	***x***	***y***	***z***	**U_{iso} ($Å^2$)**	**Site occupancy factor**
Mg	4*a*	0	0	0	0.0061(5)	0.183
Mn	4*a*	0	0	0	0.0061(5)	0.211
Fe	4*a*	0	0	0	0.0061(5)	0.196
Co	4*a*	0	0	0	0.0061(5)	0.214
Ni	4*a*	0	0	0	0.0061(5)	0.197
O	4*b*	0.5	0.5	0.5	0.0085(3)	1.0
**R_{wp} 4.70%, GOF 2.14**						

Refinement details based on NPD data collected on POWGEN at ORNL

Space group *Fm*-3*m*						
a= 4.2831(1) Å, volume = 78.5737(8) $Å^3$						
**Atom**	**Wyckoff site**	***x***	***y***	***z***	**B_{iso} ($Å^2$)**	**Site occupancy factor**
Mg	4*a*	0	0	0	0.475(15)	0.20
Mn	4*a*	0	0	0	0.475(15)	0.20
Fe	4*a*	0	0	0	0.475(15)	0.20
Co	4*a*	0	0	0	0.475(15)	0.20
Ni	4*a*	0	0	0	0.475(15)	0.20
O	4*b*	0.5	0.5	0.5	0.612(15)	1.0
**R_{wp} 10.4%**						

Table S7.

Structural refinement details of FeO-HEO obtained from XRD and NPD data.

REFERENCES AND NOTES

1. C. M. Rost, E. Sachet, T. Borman, A. Moballegh, E. C. Dickey, D. Hou, J. L. Jones, S. Curtarolo, J.-P. Maria, Entropy-stabilized oxides. *Nat. Commun.* **6**, 8485 (2015).

2. N. Dragoe, D. J. S. Bérardan, Order emerging from disorder. *Science* **366**, 573–574 (2019).

3. C. Oses, C. Toher, S. Curtarolo, High-entropy ceramics. *Nat. Rev. Mater.* **5**, 295–309 (2020).

4. C. M. Rost, Z. Rak, D. W. Brenner, J. P. Maria, Local structure of the $Mg_xNi_xCo_xCu_xZn_xO$ (x = 0.2) entropy-stabilized oxide: An EXAFS study. *J. Am. Ceram. Soc.* **100**, 2732–2738 (2017).

5. W. Y. Ching, Y. N. Xu, K. W. Wong, Ground-state and optical-properties of Cu_2O and CuO crystals. *Phys. Rev. B.* **40**, 7684–7695 (1989).

6. M. Topsakal, S. Cahangirov, E. Bekaroglu, S. Ciraci, First-principles study of zinc oxide honeycomb structures. *Phys. Rev. B.* **80**, 235119 (2009).

7. A. Sarkar, B. Breitung, H. Hahn, High entropy oxides: The role of entropy, enthalpy and synergy. *Scr. Mater.* **187**, 43–48 (2020).

8. M. Fracchia, M. Coduri, M. Manzoli, P. Ghigna, U. A. Tamburini, Is configurational entropy the main stabilizing term in rock-salt $Mg_{0.2}Co_{0.2}Ni_{0.2}Cu_{0.2}Zn_{0.2}O$ high entropy oxide? *Nat. Commun.* **13**, 2977 (2022).

9. J. Gild, M. Samiee, J. L. Braun, T. Harrington, H. Vega, P. E. Hopkins, K. Vecchio, J. Luo, High-entropy fluorite oxides. *J. Eur. Ceram. Soc.* **38**, 3578–3584 (2018).

10. K. P. Chen, X. T. Pei, L. Tang, H. R. Cheng, Z. M. Li, C. W. Li, X. W. Zhang, L. A. An, A five-component entropy-stabilized fluorite oxide. *J. Eur. Ceram. Soc.* **38**, 4161–4164 (2018).

11. S. C. Jiang, T. Hu, J. Gild, N. X. Zhou, J. Y. Nie, M. D. Qin, T. Harrington, K. Vecchio, J. Luo, A new class of high-entropy perovskite oxides. *Scr. Mater.* **142**, 116–120 (2018).

12. M. Biesuz, S. Fu, J. Dong, A. Jiang, D. Y. Ke, Q. Xu, D. G. Zhu, M. Bortolotti, M. J. Reece, C. F. Hu, S. Grasso, High entropy $Sr((Zr_{0.94}Y_{0.06})_{0.2})Sn_{0.2}Ti_{0.2}Hf_{0.2}Mn_{0.2})O_{3-x}$ perovskite synthesis by reactive spark plasma sintering. *J. Asian Ceramic Soc.* **7**, 127–132 (2019).

13. R. Witte, A. Sarkar, R. Kruk, B. Eggert, R. A. Brand, H. Wende, H. Hahn, High-entropy oxides: An emerging prospect for magnetic rare-earth transition metal perovskites. *Phys. Rev. Mater.* **3**, 034406 (2019).

14. J. Dabrowa, M. Stygar, A. Mikula, A. Knapik, K. Mroczka, W. Tejchman, M. Danielewski, M. Martin, Synthesis and microstructure of the $(Co,Cr,Fe,Mn,Ni)_3O_4$ high entropy oxide characterized by spinel structure. *Mater. Lett.* **216**, 32–36 (2018).

15. A. Q. Mao, F. Quan, H. Z. Xiang, Z. G. Zhang, K. Kuramoto, A. L. Xia, Facile synthesis and ferrimagnetic property of spinel $(CoCrFeMnNi)_3O_4$ high-entropy oxide nanocrystalline powder. *J. Mol. Struct.* **1194**, 11–18 (2019).

16. A. Q. Mao, H. Z. Xiang, Z. G. Zhang, K. Kuramoto, H. Zhang, Y. G. Jia, A new class of spinel high-entropy oxides with controllable magnetic properties. *J. Magn. Magn. Mater.* **497**, 165884 (2020).

17. K. P. Tseng, Q. Yang, S. J. McCormack, W. M. Kriven, High-entropy, phase-constrained, lanthanide sesquioxide. *J. Am. Ceram. Soc.* **103**, 569–576 (2020).

18. F. Li, L. Zhou, J. X. Liu, Y. C. Liang, G. J. Zhang, High-entropy pyrochlores with low thermal conductivity for thermal barrier coating materials. *J. Adv. Ceram.* **8**, 576–582 (2019).

19. Z. F. Zhao, H. M. Xiang, F. Z. Dai, Z. J. Peng, Y. C. Zhou, $(La_{0.2}Ce_{0.2}Nd_{0.2}Sm_{0.2}Eu_{0.2})_2Zr_2O_7$: A novel high-entropy ceramic with low thermal conductivity and sluggish grain growth rate. *J. Mater. Sci. Technol.* **35**, 2647–2651 (2019).

20. A. Sarkar, L. Velasco, D. Wang, Q. Wang, G. Talasila, L. de Biasi, C. Kübel, T. Brezesinski, S. S. Bhattacharya, H. Hahn, High entropy oxides for reversible energy storage. *Nat. Commun.* **9**, 3400 (2018).

21. C. Zhao, F. Ding, Y. Lu, L. Chen, Y. S. Hu, High-entropy layered oxide cathodes for sodium-ion batteries. *Angew. Chem. Int. Ed.* **59**, 264–269 (2020).

22. X. F. Luo, J. Patra, W. T. Chuang, T. X. Nguyen, J. M. Ting, J. Li, C. W. Pao, J. K. Chang, Charge-discharge mechanism of high-entropy co-free spinel oxide toward Li^+ storage examined using operando quick-scanning x-ray absorption spectroscopy. *Adv. Sci.* **9**, 2201219 (2022).

23. T. Y. Li, Y. G. Yao, Z. N. Huang, P. F. Xie, Z. Y. Liu, M. H. Yang, J. L. Gao, K. Z. Zeng, A. H. Brozena, G. Pastel, M. L. Jiao, Q. Dong, J. Q. Dai, S. K. Li, H. Zong, M. F. Chi, J. Luo, Y. F. Mo, G. F. Wang, C. Wang, R. Shahbazian-Yassar, L. B. Hu, Author correction: Denary oxide nanoparticles as highly stable catalysts for methane combustion. *Nat. Catal.* **4**, 439–439 (2021).

24. H. Xu, Z. Zhang, J. Liu, C.-L. Do-Thanh, H. Chen, S. Xu, Q. Lin, Y. Jiao, J. Wang, Y. Wang, Entropy-stabilized single-atom Pd catalysts via high-entropy fluorite oxide supports. *Nat. Commun.* **11**, 3908 (2020).

25. D. Y. Feng, Y. B. Dong, L. L. Zhang, X. Ge, W. Zhang, S. Dai, Z. A. Qiao, Holey lamellar high-entropy oxide as an ultra-high-activity heterogeneous catalyst for solvent-free aerobic oxidation of benzyl alcohol. *Angew. Chem. Int. Ed.* **59**, 19503–19509 (2020).

26. D. Berardan, S. Franger, A. K. Meena, N. Dragoe, Room temperature lithium superionic conductivity in high entropy oxides. *J. Mater. Chem. A* **4**, 9536–9541 (2016).

27. N. Osenciat, D. Berardan, D. Dragoe, B. Leridon, S. Hole, A. K. Meena, S. Franger, N. Dragoe, Charge compensation mechanisms in Li-substituted high-entropy oxides and influence on Li superionic conductivity. *J. Am. Ceram. Soc.* **102**, 6156–6162 (2019).

28. J. J. Zhang, J. Q. Yan, S. Calder, Q. Zheng, M. A. McGuire, D. L. Abernathy, Y. Ren, S. H. Lapidus, K. Page, H. Zheng, J. W. Freeland, J. D. Budai, R. P. Hermann, Long-range antiferromagnetic order in a rocksalt high entropy oxide. *Chem. Mater.* **31**, 3705–3711 (2019).

29. M. P. Jimenez-Segura, T. Takayama, D. Berardan, A. Hoser, M. Reehuis, H. Takagi, N. Dragoe, Long-range magnetic ordering in rocksalt-type high-entropy oxides. *Appl. Phys. Lett.* **114**, 122401 (2019).

30. Y. F. Sun, S. Dai, High-entropy materials for catalysis: A new frontier. *Sci. Adv.* **7**, eabg1600 (2021).

31. A. Sarkar, R. Kruk, H. Hahn, Magnetic properties of high entropy oxides. *Dalton Trans.* **50**, 1973–1982 (2021).

32. G. M. Tomboc, X. D. Zhang, S. Choi, D. Kim, L. Y. S. Lee, K. Lee, Stabilization, characterization, and electrochemical applications of high-entropy oxides: Critical assessment of crystal phase-properties relationship. *Adv. Funct. Mater.* **32**, 2205142 (2022).

33. S. Akrami, P. Edalati, M. Fuji, K. Edalati, High-entropy ceramics: Review of principles, production and applications. *Mater. Sci. Eng. R Rep.* **146**, 100644 (2021).

34. J. Chen, W. X. Liu, J. X. Liu, X. L. Zhang, M. Z. Yuan, Y. L. Zhao, J. J. Yan, M. Q. Hou, J. Y. Yan, M. Kunz, N. Tamura, H. Z. Zhang, Z. L. Yin, Stability and compressibility of cation-doped high-entropy oxide $MgCoNiCuZnO_5$. *J. Phys. Chem. C* **123**, 17735–17744 (2019).

35. A. L. F. Cardoso, C. P. F. Perdomo, R. H. G. A. Kiminami, R. F. K. Gunnewiek, Enhancing the stabilization of nanostructured rocksalt-like high entropy oxide by Gd addition. *Mater. Lett.* **285**, 129175 (2021).

36. K. C. Pitike, K. C. Santosh, M. Eisenbach, C. A. Bridges, V. R. Cooper, Predicting the phase stability of multicomponent high-entropy compounds. *Chem. Mater.* **32**, 7507–7515 (2020).

37. L. Broussard, Disproportionation of wustite. *J. Phys. Chem.* **73**, 1848–1854 (1969).

38. F. Schrettle, C. Kant, P. Lunkenheimer, F. Mayr, J. Deisenhofer, A. Loidl, Wüstite: Electric, thermodynamic and optical properties of FeO. *Eur. Phys. J. B.* **85**, 164 (2012).

39. B. P. Pichon, O. Gerber, C. Lefevre, I. Florea, S. Fleutot, W. Baaziz, M. Pauly, M. Ohlmann, C. Ulhaq, O. Ersen, V. Pierron-Bohnes, P. Panissod, M. Drillon, S. Begin-Colin, Microstructural and magnetic investigations of wüstite-spinel core-shell cubic-shaped nanoparticles. *Chem. Mater.* **23**, 2886–2900 (2011).

40. B. Musico, Q. Wright, T. Z. Ward, A. Grutter, E. Arenholz, D. Gilbert, D. Mandrus, V. Keppens, Tunable magnetic ordering through cation selection in entropic spinel oxides. *Phys. Rev. Mater.* **3**, 104416 (2019).

41. Y. Ma, Y. Ma, Q. Wang, S. Schweidler, M. Botros, T. Fu, H. Hahn, T. Brezesinski, B. Breitung, High-entropy energy materials: Challenges and new opportunities. *Energ. Environ. Sci.* **14**, 2883–2905 (2021).

42. M. Hermanek, R. Zboril, M. Mashlan, L. Machala, O. Schneeweiss, Thermal behaviour of iron(II) oxalate dihydrate in the atmosphere of its conversion gases. *J. Mater. Chem.* **16**, 1273–1280 (2006).

43. M. C. Puerta, P. Valerga, Thermal decomposition of a natural manganese dioxide: A laboratory experiment for undergraduate students. *J. Chem. Educ.* **67**, 344–346 (1990).

44. B. D. Desai, J. B. Fernandes, V. N. K. Dalal, Manganese dioxide—A review of a battery chemical Part II. Solid state and electrochemical properties of manganese dioxides. *J. Power Sources* **16**, 1–43 (1985).

45. S. Sasaki, K. Fujino, Y. Takéuchi, X-ray determination of electron-density distributions in oxides, MgO, MnO, CoO, and NiO, and atomic scattering factors of their constituent atoms. *Proc. Jpn. Acad. Ser. B. Phys.* **55**, 43–48 (1979).

46. A. Jay, K. Andrews, Note on oxide systems pertaining to steel-making furnace slags: FeO-MnO, FeO-MgO, CaO-MnO, MgO-MnO. *J. Iron. Steel. Inst.* **152**, 15–18 (1945).

47. Q. H. Bian, S. J. Lei, K. H. Zhao, Q. Y. Tu, L. Zhao, L. H. Rao, Y. H. Xiao, B. C. Cheng, Isomorphous substitution synthesis and photoelectric properties of spinel AgInSnS 4 nanosheets. *Chem. Mater.* **32**, 9713–9720 (2020).

48. D. Bérardan, A. K. Meena, S. Franger, C. Herrero, N. Dragoe, Controlled Jahn-Teller distortion in (MgCoNiCuZn)O-based high entropy oxides. *J. Alloys Compd.* **704**, 693–700 (2017).

49. Y. Ren, X. B. Zuo, Synchrotron x-ray and neutron diffraction, total scattering, and small-angle scattering techniques for rechargeable battery research. *Small Methods* **2**, 1800064 (2018).

50. C. J. Chen, R. K. Chiang, H. Y. Lai, C. R. Lin, Characterization of monodisperse Wüstite nanoparticles following partial oxidation. *J. Phys. Chem. C* **114**, 4258–4263 (2010).

51. C. J. Chen, R. K. Chiang, S. Kamali, S. L. Wang, Synthesis and controllable oxidation of monodisperse cobalt-doped Wüstite nanoparticles and their core–shell stability and exchange-bias stabilization. *Nanoscale* **7**, 14332–14343 (2015).

52. X. L. Sun, N. F. Huls, A. Sigdel, S. H. Sun, Tuning exchange bias in core/shell FeO/Fe_3O_4 nanoparticles. *Nano Lett.* **12**, 246–251 (2012).

53. Z. C. Yan, S. FitzGerald, T. M. Crawford, O. T. Mefford, Oxidation of wüstite rich iron oxide nanoparticles via post-synthesis annealing. *J. Magn. Magn. Mater.* **539**, 168405 (2021).

54. A. Ullrich, N. Rolle, S. Horn, From wustite to hematite: Thermal transformation of differently sized iron oxide nanoparticles in air. *J. Nanopart. Res.* **21**, 168 (2019).

55. K. M. Nam, J. H. Shim, D.-W. Han, H. S. Kwon, Y.-M. Kang, Y. Li, H. Song, W. S. Seo, J. T. Park, Syntheses and characterization of wurtzite CoO, rocksalt CoO, and spinel Co3O4 nanocrystals: Their interconversion and tuning of phase and morphology. *Chem. Mater.* **22**, 4446–4454 (2010).

56. Y. Zhang, T. T. Zuo, Z. Tang, M. C. Gao, K. A. Dahmen, P. K. Liaw, Z. P. Lu, Microstructures and properties of high-entropy alloys. *Prog. Mater. Sci.* **61**, 1–93 (2014).

57. C. W. Chou, S. J. Chu, H. J. Chiang, C. Y. Huang, C. J. Lee, S. R. Sheen, T. P. Perng, C. T. Yeh, Temperature-programmed reduction study on calcination of nano-palladium. *J. Phys. Chem. B* **105**, 9113–9117 (2001).

58. J. A. Rodriguez, J. C. Hanson, A. I. Frenkel, J. Y. Kim, M. Perez, Experimental and theoretical studies on the reaction of H2with NiO: Role of O vacancies and mechanism for oxide reduction. *J. Am. Chem. Soc.* **124**, 346–354 (2002).

59. A. Y. Khodakov, W. Chu, P. Fongarland, Advances in the development of novel cobalt Fischer-Tropsch catalysts for synthesis of long-chain hydrocarbons and clean fuels. *Chem. Rev.* **107**, 1692–1744 (2007).

60. Y. H. Hu, Solid-solution catalysts for CO_2 reforming of methane. *Catal. Today* **148**, 206–211 (2009).

61. M. Newville, Fundamentals of XAFS. *Rev. Mineral. Geochem.* **78**, 33–74 (2014).

62. A. Kuzmin, N. Mironova, J. Purans, A. Sazonov, EXAFS and XANES studies of $Co_xMg_{1-x}O$ solid solutions using a laboratory EXAFS spectrometer. *Phys. Status Solidi A* **135**, 133–141 (1993).

63. N. Li, R. G. Hadt, D. Hayes, L. X. Chen, D. G. Nocera, Detection of high-valent iron species in alloyed oxidic cobaltates for catalysing the oxygen evolution reaction. *Nat. Commun.* **12**, 4218 (2021).

64. C. A. McCammon, D. C. Price, Mössbauer spectra of Fe_x O ($x > 0.95$). *Phys. Chem. Miner.* **11**, 250–254 (1985).

65. M. Herlitschke, A. L. Tchougreeff, A. V. Soudackov, B. Klobes, L. Stork, R. Dronskowski, R. P. Hermann, Magnetism and lattice dynamics of FeNCN compared to FeO. *New J. Chem.* **38**, 4670–4677 (2014).

66. G. Shirane, D. E. Cox, S. L. Ruby, Mössbauer study of isomer shift, quadrupole interaction, and hyperfine field in several oxides containing Fe^{57}. *Phys. Rev.* **125**, 1158–1165 (1962).

67. C. Gohy, A. Gerard, F. Grandjean, Mössbauer study of wustite and mangano-wustite. *Phys. Status Solidi A* **74**, 583–591 (1982).

68. R. Hermann, Mössbauer spectroscopy, in *Handbook of Solid State Chemistry,* R. Dronskowski, S. Kikkawa, A. Stein, Eds. (John Wiley & Sons, ed. 1st, 2017), vol. 3, pp. 443.

69. B. Hentschel, Stoichiometric FeO as metastable intermediate of the decomposition of Wustite at 225 °C. *Zeitschrift für Naturforschung A* **25**, 1996–1997 (1970).

70. B. A. Frandsen, K. A. Petersen, N. A. Ducharme, A. G. Shaw, E. J. Gibson, B. Winn, J. Q. Yan, J. J. Zhang, M. E. Manley, R. P. Hermann, Spin dynamics and a nearly continuous magnetic phase transition in an entropy-stabilized oxide antiferromagnet. *Phys. Rev. Mater.* **4**, 074405 (2020).

71. K. C. Pitike, A. Macias, M. Eisenbach, C. A. Bridges, V. R. Cooper, Computationally accelerated discovery of high entropy pyrochlore oxides. *Chem. Mater.* **34**, 1459–1472 (2022).

72. D. Stull, H. Prophet, *JANAF Thermochemical Tables* (NBS National Standard Reference Data Series (NSRDS), US National Bureau of Standards, ed. Second Edition, 1971).

73. K. Reuter, M. Scheffler, Composition, structure, and stability of RuO_2(110) as a function of oxygen pressure. *Phys. Rev. B.* **65**, 035406 (2001).

74. J. Rodriguez-Carvajal, in *Satellite meeting on powder diffraction of the XV congress of the IUCr*. (Toulouse, France, 1990), pp. 127.

75. B. Ravel, M. Newville, ATHENA, ARTEMIS, HEPHAESTUS: Data analysis for X-ray absorption spectroscopy using IFEFFIT. *J. Synchrotron. Rad.* **12**, 537–541 (2005).

76. G. Kresse, J. Furthmüller, Efficiency of ab-initio total energy calculations for metals and semiconductors using a plane-wave basis set. *Comput. Mater. Sci.* **6**, 15–50 (1996).

77. G. Kresse, J. Furthmüller, Efficient iterative schemes for ab initio total-energy calculations using a plane-wave basis set. *Phys. Rev. B.* **54**, 11169–11186 (1996).

78. J. P. Perdew, A. Ruzsinszky, G. I. Csonka, O. A. Vydrov, G. E. Scuseria, L. A. Constantin, X. Zhou, K. Burke, Restoring the density-gradient expansion for exchange in solids and surfaces. *Phys. Rev. Lett.* **100**, 136406 (2008).

79. P. E. Blöchl, Projector augmented-wave method. *Phys. Rev. B.* **50**, 17953–17979 (1994).

80. G. Kresse, D. Joubert, From ultrasoft pseudopotentials to the projector augmented-wave method. *Phys. Rev. B.* **59**, 1758–1775 (1999).

81. S. L. Dudarev, G. A. Botton, S. Y. Savrasov, C. Humphreys, A. P. Sutton, Electron-energy-loss spectra and the structural stability of nickel oxide: An LSDA+U study. *Phys. Rev. B.* **57**, 1505–1509 (1998).

82. J. K. Burdett, G. D. Price, S. L. Price, Role of the crystal-field theory in determining the structures of spinels. *J. Am. Chem. Soc.* **104**, 92–95 (1982).

83. B. H. Toby, R factors in Rietveld analysis: How good is good enough? *Powder Diffr.* **21**, 67–70 (2006).

84. A. E. Martell, R. M. Smith, *Other organic ligands* (Critical Stability Constants, Springer, ed. 1, 2013).

85. R. M. Smith, A. E. Martell, Critical stability constants, Enthalpies and Entropies for the Formation of metal complexes of aminopolycarboxylic Acids and carboxylic acids. *Sci. Total Environ.* **64**, 125–147 (1987).

86. J. Lin, S. Jacosben, W. Sturhahn, J. Jackson, J. Zhao, C. Yoo, Sound velocities of ferropericlase in the Earth's lower mantle. *Geophys. Res. Lett.* **33**, 10.1029/2006GL028099 (2006).
